# Comparison of the efficacy of platelet-rich plasma versus corticosteroid in the treatment of adhesive capsulitis: a systematic review and meta-analysis based on randomized controlled trials

**DOI:** 10.3389/fmed.2026.1766836

**Published:** 2026-02-05

**Authors:** Chang Xu, Ziyue Wang, Junru Tan, Ningning Zhou, Qingling Zhong, Yajun Chen, Rilong Huang

**Affiliations:** 1 Department of Acupuncture, Anhui University of Chinese Medicine, Hefei, Anhui, China; 2 Department of Orthopedics and Traumatology, The First Affiliated Hospital of Zhejiang Chinese Medical University, Hangzhou, China; 3 Department of Acupuncture, The First Affiliated Hospital of Anhui University of Chinese Medicine, Hefei, Anhui, China

**Keywords:** adhesive capsulitis, frozen shoulder, platelet-rich plasma, corticosteroid, meta-analysis

## Abstract

**Background:**

Adhesive capsulitis (AC) often impairs patients’ quality of life due to shoulder pain and restricted joint mobility. Intra-articular shoulder injection is a profound conservative treatment modality. Existing randomized controlled trials (RCTs) have reported conflicting results regarding the efficacy of platelet-rich plasma (PRP) versus corticosteroid (CS) injections. Therefore, a meta-analysis of the relevant RCTs is warranted.

**Methods:**

A systematic search was conducted across four databases (PubMed, Embase, the Cochrane Library, and Web of Science) for articles published from their inception to September 15, 2025. RCTs comparing the efficacy of PRP versus CS injections for the treatment of AC were included. The primary outcomes were the Visual Analog Scale (VAS) score and the Disabilities of the Arm, Shoulder and Hand (DASH) score. Secondary outcomes included range of motion (ROM): abduction, flexion, external rotation, and internal rotation.

**Results:**

This meta-analysis included a total of 13 studies involving 1,056 patients with AC. Among them, 531 patients were allocated to the PRP group and 525 to the CS group. No statistically significant differences were observed between the two groups in the VAS and DASH scores at 1 month, VAS score at 3 months, or flexion ROM. However, compared with the CS group, the PRP group demonstrated significantly superior outcomes in the VAS score at 6 months, DASH scores at 3 and 6 months, as well as in abduction, external rotation, and internal rotation. Specifically, significant differences were observed in: the 6-month VAS score (MD: –1.84, 95% CI: −2.57 to −1.10, *p* < 0.00001), the 3-month DASH score (MD: –5.88, 95% CI: −9.72 to −2.03, *p* = 0.003), the 6-month DASH score (MD: –14.42, 95% CI: −16.35 to −12.49, *p* < 0.00001), abduction (MD: 11.90, 95% CI: 2.23 to 21.57, *p* = 0.02), external rotation (MD: 8.39, 95% CI: 1.39 to 15.40, *p* = 0.02), and internal rotation (MD: 10.04, 95% CI: 8.80 to 11.29, *p* < 0.00001).

**Conclusion:**

Compared with CS, PRP for AC demonstrated significant advantages in pain relief, functional improvement, and range of motion recovery at the 6-month follow-up. However, the two treatments showed comparable efficacy in terms of pain relief at the 1- to 3-month follow-ups and functional improvement at the 1-month follow-up.

**Systematic review registration:**

https://www.crd.york.ac.uk/PROSPERO/, CRD420251156731.

## 1 Introduction

Adhesive Capsulitis (AC), also known as “frozen shoulder,” is an idiopathic glenohumeral joint disorder characterized pathologically by capsular thickening and contracture, with clinical manifestations including peri-shoulder pain and progressive limitation of range of motion (1). The prevalence of AC in the general population is approximately 2–5%, and it is more common in women over 40 years of age (2). AC is typically self-limiting, with most patients experiencing spontaneous symptom resolution within 1–2 years without intervention (3). However, emerging evidence suggests that without standardized treatment, some patients may suffer from persistent shoulder dysfunction and pain, significantly impairing daily activities (4).

The current treatment goals for AC primarily include pain relief, improvement in joint range of motion, and reduction of functional impairment, thereby enhancing patients’ quality of life. Intra-articular shoulder injection serves as an important conservative treatment modality between physical therapy and surgical intervention, and is widely used in clinical practice. Among these interventions, CS injection is often considered a first-line treatment option due to its potent anti-inflammatory and analgesic effects (5). Multiple studies have confirmed that CS can significantly alleviate pain and restore joint function in the short term (6, 7). However, the therapeutic effect is often difficult to sustain over the long term, with a potential decline in efficacy or recurrence of symptoms over time (8, 9). Furthermore, repeated CS injections carry potential risks, such as cartilage damage and reduced tendon structural strength (10, 11). In recent years, PRP has garnered considerable attention as a regenerative medicine therapy in the treatment of musculoskeletal disorders (12). By releasing high concentrations of growth factors, PRP modulates local inflammatory responses and promotes tissue healing and regeneration (13). This suggests that PRP may intervene at a more fundamental level in the pathological process of AC and has the potential to provide more sustained clinical benefits.

Although several RCTs have compared the efficacy of PRP with CS in the treatment of AC, the results remain inconsistent. Therefore, this study aims to conduct a meta-analysis based on relevant RCTs to comprehensively evaluate whether there is a difference in efficacy between the two treatments from three dimensions: pain level, shoulder joint function, and range of motion improvement. The findings are expected to provide high-quality evidence-based medical evidence for clinical decision-making.

## 2 Materials and methods

This study adhered to the Meta-analysis Of Observational Studies in Epidemiology (MOOSE) guidelines and followed the Preferred Reporting Items for Systematic Reviews and Meta-Analyses (PRISMA) statement (14). The review protocol has been registered in the PROSPERO database (Registration No. CRD420251156731).

### 2.1 Literature search

The two authors conducted a search using keywords such as “Adhesive capsulitis,” “Frozen shoulder,” “Platelet-rich plasma,” and “Corticosteroid” in human subjects from the inception dates of PubMed, Embase, Web of Science, and Cochrane Library until September 15, 2025. The specific search strategy can be found in Supplementary file S1.

### 2.2 Inclusion and exclusion criteria

The inclusion criteria are: (1) RCTs comparing the efficacy of PRP versus CS in the treatment of AC; (2) studies reporting at least one of the following outcome measures: VAS, DASH, or range of motion including abduction, flexion, external rotation, and internal rotation. The exclusion criteria are: (1) non-RCTs, reviews, letters, commentaries, case reports, conference abstracts, or other types of publications; (2) studies that did not provide sufficient data for analysis; (3) studies in which the sample size of any group was fewer than 20 patients.

### 2.3 Data extraction

Two researchers independently reviewed the abstracts and full texts to identify eligible studies, resolving any disagreements through consultation with a third researcher. The two researchers independently extracted data using a standardized data extraction form, covering key information such as the authors, publication year, study type, comparisons, number of patients, age, gender, follow-up time, and outcome measures. For continuous data presented as median and interquartile range in the included studies, we converted them to mean ± standard deviation using validated mathematical methods.

### 2.4 Outcome measures

We employed the VAS and the DASH scores as measures of pain intensity and functional status, respectively. Shoulder range of motion was assessed based on abduction, flexion, external rotation, and internal rotation angles. The VAS score ranges from 0 to 10, with higher scores indicating greater pain intensity (15). The DASH score ranges from 0 to 100, with lower scores representing better functional ability (16). For the VAS and DASH, outcomes were assessed at the 1-month, 3-month, and 6-month follow-ups, respectively. ROM measurements were collected and evaluated only at the final follow-up.

### 2.5 Assessment of risk of bias and quality of evidence

**Figure 1:**
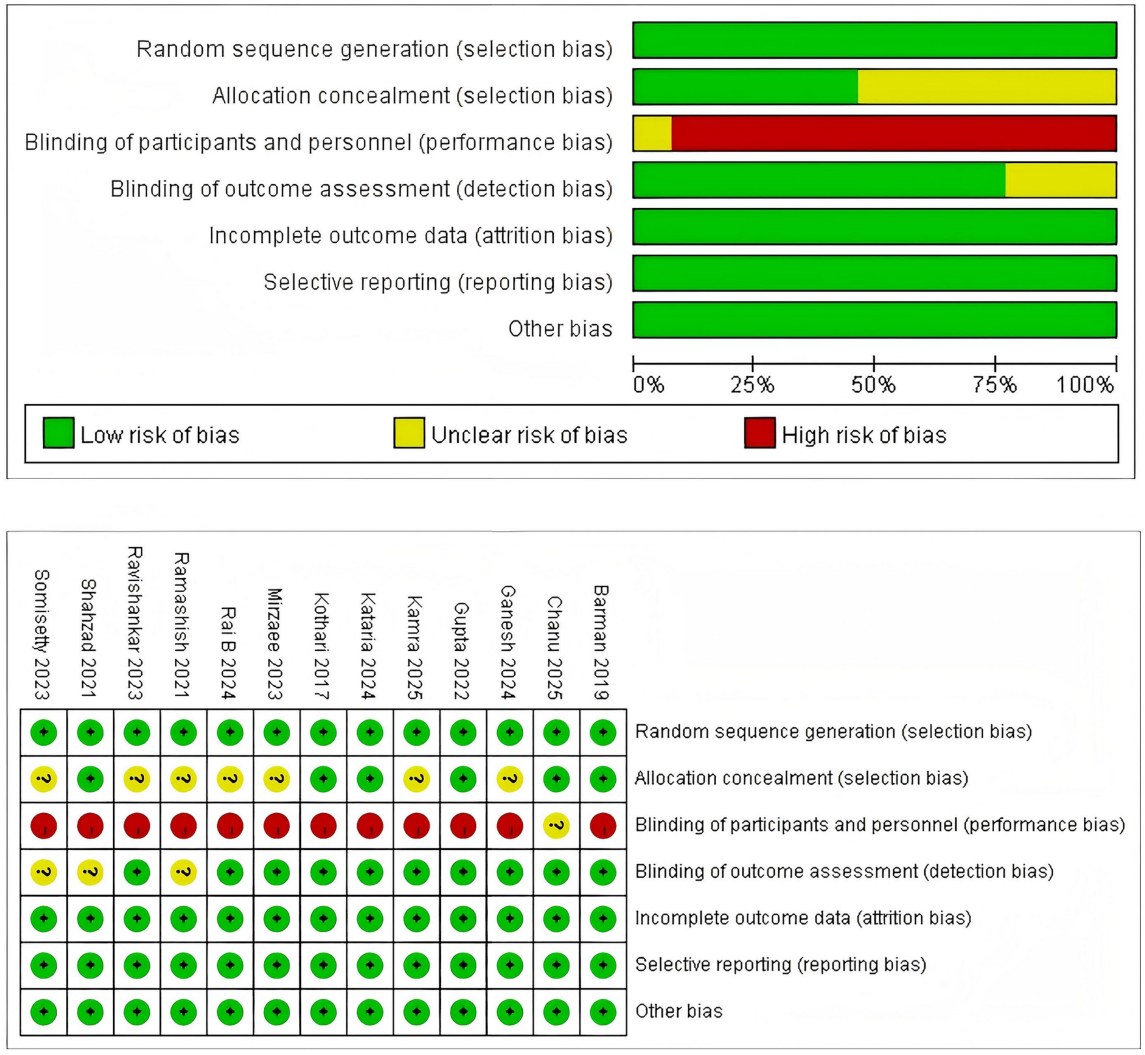
Risk of bias assessment of included RCTs.

The quality assessment in this study was jointly completed by two authors, and any disagreements were resolved by a third reviewer. The quality of RCTs was evaluated based on seven factors using the Cochrane RoB 2 (17). Trials were categorized as low, high, or moderate quality: If there was a high risk of bias in randomization or allocation concealment, the trial was classified as low quality, regardless of other criteria; if both randomization and allocation concealment were at low risk, and other criteria were either at low risk or unclear, the trial was considered high quality; if it did not meet any high-risk or low-risk criteria, it was deemed moderate quality. The 13 included RCTs comprised one high-quality study and 12 medium-quality studies. Detailed information on the quality assessment is provided in Figure 1.

### 2.6 Data analysis

Data analysis was conducted using Review Manager 5.4 and Stata 15.1. The mean difference (MD) was employed for continuous variables. All indicators were represented using a 95% confidence interval (CI). Heterogeneity among outcome indicators was assessed using the I^2^. Significant heterogeneity was indicated by an I^2^ value greater than 50%. If *I*^2^ < 50%, heterogeneity among studies was considered insignificant, and a fixed-effects model was employed. If *I*^2^ > 50%, significant heterogeneity among studies was indicated, and a random-effects model was adopted. To explore the sources of heterogeneity, subgroup analyses (based on CS dosage and sex differences) or a “leave-one-out” analysis (LOO) were subsequently performed. Statistical significance was defined as *p* < 0.05. Furthermore, publication bias was assessed using funnel plots (Supplementary file S4), supplemented by Egger’s test (Supplementary file S5) and Begg’s test (Supplementary file S6). A *p*-value > 0.05 for both tests was considered indicative of no significant bias; otherwise, significant publication bias was deemed to be present.

## 3 Results

### 3.1 Search results

**Figure 2:**
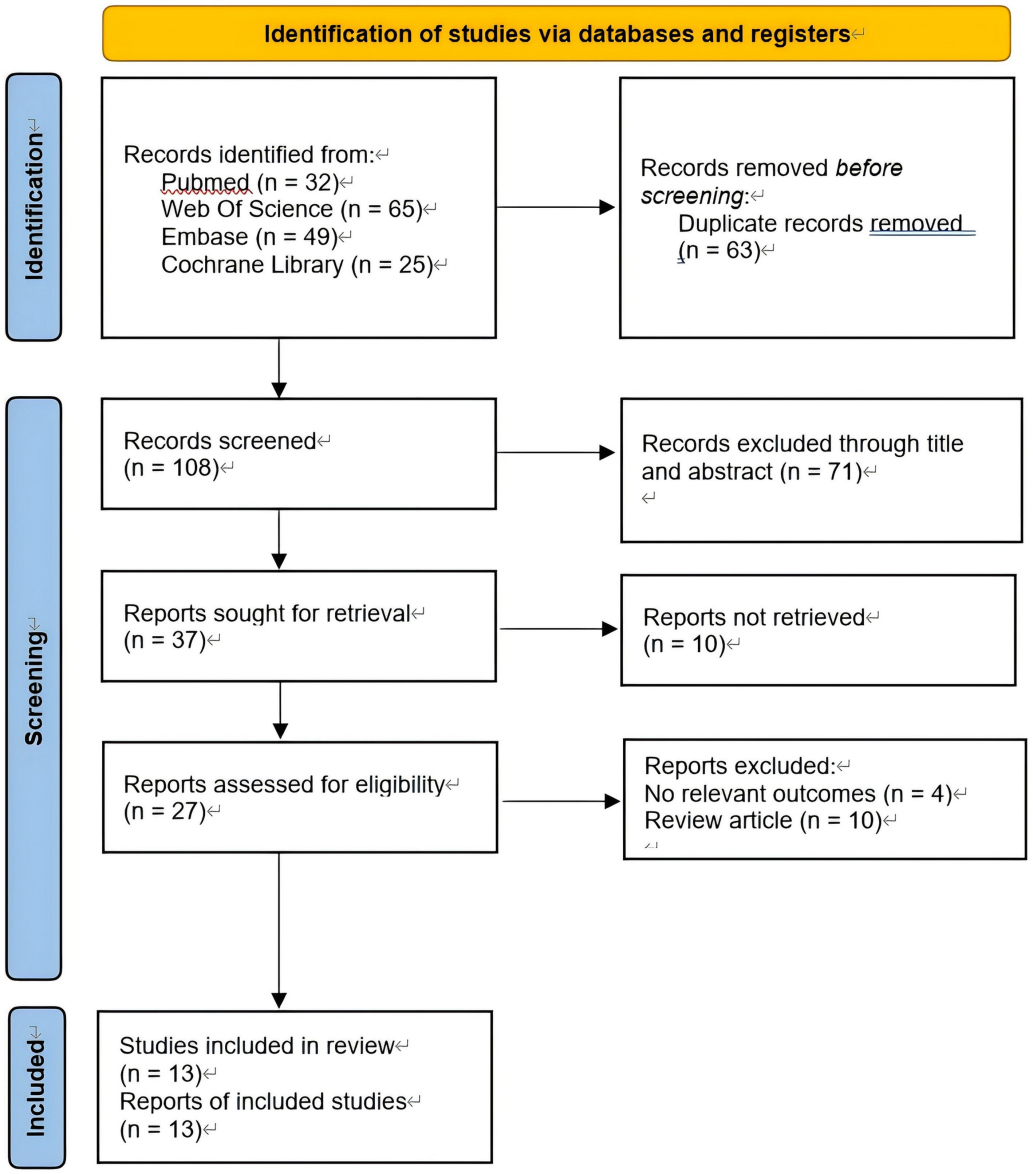
PRISMA flow diagram for a summary of the search process.

A total of 171 articles were retrieved through database searches. After removing duplicates, 108 articles remained for title and abstract screening. Among these, 37 articles underwent full-text review, and 24 were excluded due to not meeting the inclusion criteria. Ultimately, 13 eligible RCTs were included in this meta-analysis. All the included studies investigated the use of PRP versus CS in the treatment of AC. The specific screening and exclusion process is illustrated in Figure 2.

### 3.2 Characteristics of studies and demographic data

This meta-analysis included a total of 13 RCTs, comprising 1,056 patients. All studies were published in English. The mean age of participants across the included studies ranged from 40 to 60 years. Follow-up duration was 3 months in 5 studies, 4.5 months in 1 study, and 6 months in 7 studies. Among all patients, 531 were subjected to PRP treatment, while 525 received CS treatment. The basic characteristics and demographic data of the included studies are summarized in Table 1.

**Table 1:** Characteristics of studies and demographic data.

Study ID	Type of study	Comparisons	Number of patients	Gender (M/F)	Mean age (years)	Follow up (month)
Kothari et al. (25)	RCT	PRP 2 mL CS 2 mL	62 60	34/28 29/31	51.9 ± 10.1 52.7 ± 8.6	3
Barman et al. (44)	RCT	PRP 4 mL CS 1 mL	28 27	NA NA	NA NA	3
Yadav et al. (19)	RCT	PRP 4 mL CS 2 mL	30 30	25/35	NA NA	3
Shahzad et al. (18)	RCT	PRP 2 mL CS 2 mL	100 100	42/58 41/59	52.41 ± 2.67 53.0 ± 3.74	3
Gupta et al. (20)	RCT	PRP 2 mL CS 2 mL	30 30	25/35	47.25 ± 8.38	6
Ravishankar et al. (45)	RCT	PRP 2 mL CS 1 mL	28 28	34/22	56.04 ± 6.43 55.75 ± 7.29	4.5
Somisetty et al. (26)	RCT	PRP 4 mL CS 2 mL	34 34	15/19 20/14	58.3 ± 8.1 58.5 ± 7.7	6
Mirzaee et al. (43)	RCT	PRP 10 mL CS 1 mL	72 69	18/54 14/55	56.72 ± 8.00 60.59 ± 9.31	3
Ganesh et al. (32)	RCT	PRP 2 ml CS 1 mL	30 30	33/27	43.4	6
Kataria et al. (46)	RCT	PRP 4 mL CS 1 ml	30 30	20/10 18/12	51.0 ± 4.62 52.41 ± 4.91	6
Rai et al. (27)	RCT	PRP 5 mL CS 2 ml	30 30	14/16 17/13	48.83 ± 9.60 46.94 ± 11.98	6
Kamra et al. (21)	RCT	PRP 2 mL CS 2 mL	25 25	10/15 11/14	46.70 ± 7.64 45.80 ± 6.86	6
Chanu et al. (47)	RCT	PRP 4 mL CS 1 mL	32 32	11/21 11/21	54.75 ± 6.6 56.03 ± 5.6	6

### 3.3 Main outcomes

#### 3.3.1 VAS: 1 month

**Figure 3:**
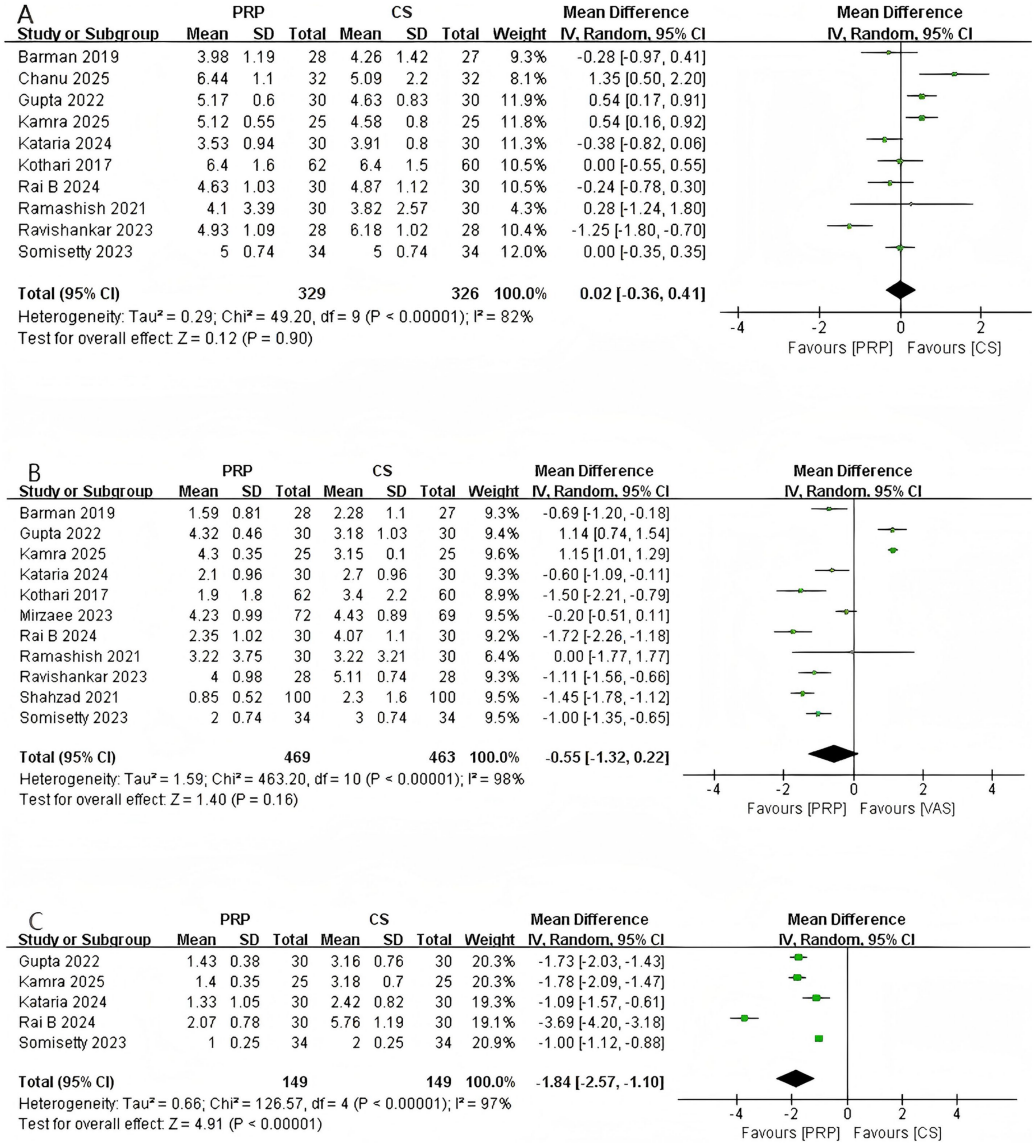
Forest plot of VAS. **(A)** Forest plot of 1-month VAS. **(B)** Forest plot of 3-month VAS. **(C)** Forest plot of 6-month VAS.

As shown in Figure 3A, 10 studies (*n* = 655) reported data on the 1-month VAS score. The pooled results indicated no significant difference between the two groups in the 1-month VAS score (MD: 0.02, 95% CI: −0.36 to 0.41, *p* = 0.90). Significant heterogeneity was observed among the studies (Chi^2^ = 42.90, *p* < 0.00001, *I*^2^ = 82%). LOO method and subgroup analyses based on steroid dosage and gender did not identify the source of heterogeneity. For further details, please refer to Supplementary file S2, S3.

#### 3.3.2 VAS: 3 month

As shown in Figure 3B, a total of 11 studies involving 932 patients reported 3-month VAS scores. The pooled results indicated no significant difference in the 3-month VAS between the two groups (MD: –0.55, 95% CI: −1.32 to 0.22, *p* = 0.16). Significant heterogeneity was observed among the studies (Chi^2^ = 463.20, *p* < 0.00001, *I*^2^ = 98%). LOO method and subgroup analysis did not identify the source of heterogeneity. For details, please refer to Supplementary file S2, S3.

#### 3.3.3 VAS: 6 month

As shown in Figure 3C, a total of five studies (*n* = 298) reported the VAS at 6 months. The pooled analysis revealed a statistically significant difference in the 6-month VAS between the two groups (MD: –1.84, 95% CI: −2.57 to −1.10, *p* < 0.00001), indicating superior sustained analgesic efficacy of PRP compared to CS injection. However, significant heterogeneity was observed among the studies (Chi^2^ = 126.57, *p* < 0.00001, *I*^2^ = 97%). LOO analysis did not identify the source of heterogeneity. Subgroup analysis indicated that heterogeneity was not observed in studies with a higher proportion of female participants (*I*^2^ = 0), whereas significant heterogeneity persisted in studies with a higher proportion of male participants (*I*^2^ = 98%). Further details are provided in Supplementary file S2, S3.

#### 3.3.4 DASH: 1 month

**Figure 4:**
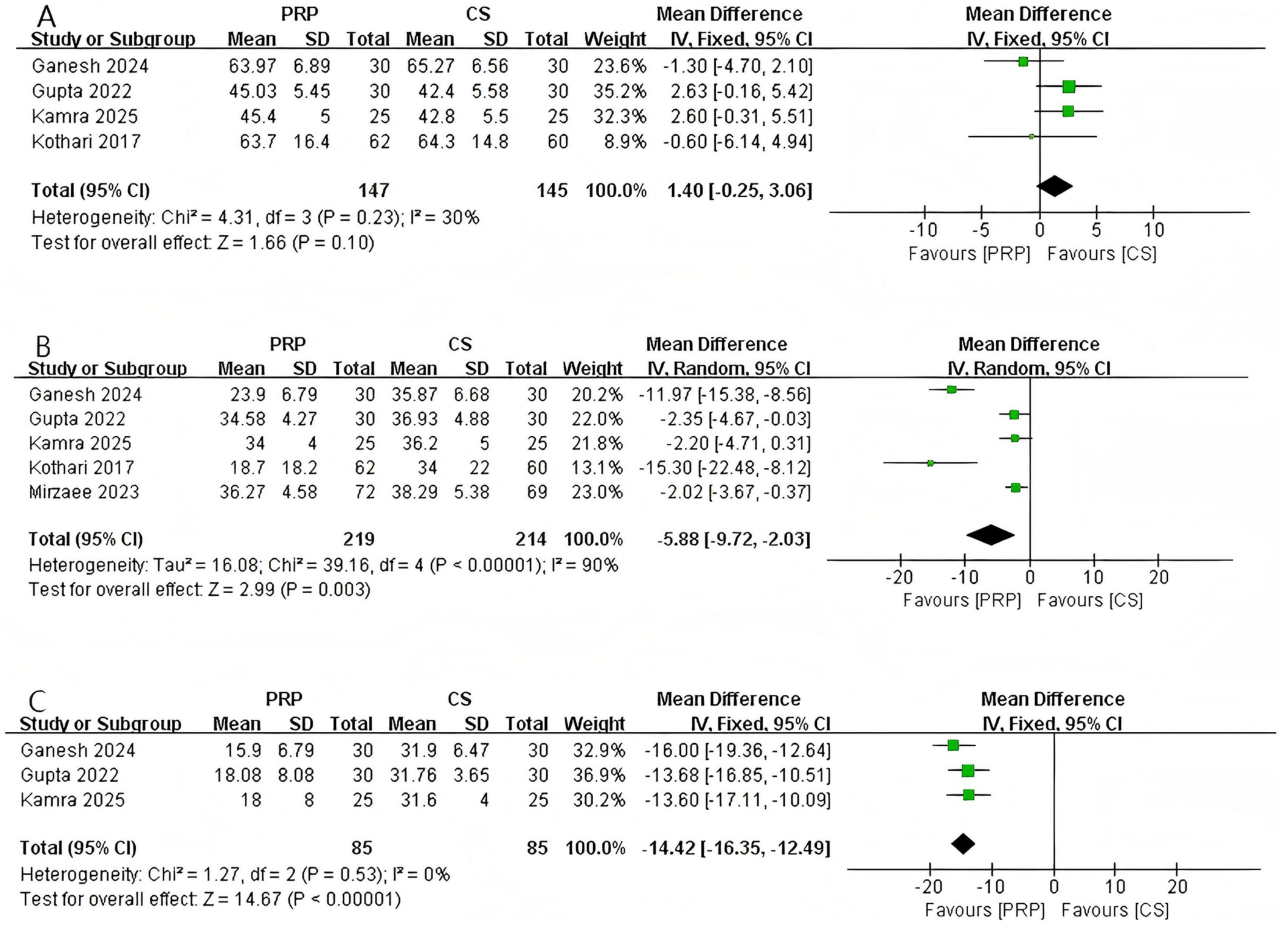
Forest plot of DASH. **(A)** Forest plot of 1-month DASH. **(B)** Forest plot of 3-month DASH. **(C)** Forest plot of 6-month DASH.

As shown in Figure 4A, 4 studies (*n* = 292) reported DASH scores at 1-month. The pooled results demonstrated no statistically significant difference between the two groups at 1-month (MD: 1.40, 95% CI: −0.25 to 3.06, *p* = 0.10). No significant heterogeneity was observed among the included studies (Chi^2^ = 4.31, *p* = 0.23, *I*^2^ = 30%).

#### 3.3.5 DASH: 3 month

A total of five studies involving 433 patients reported the DASH score at 3 months (Figure 4B). The pooled analysis revealed a statistically significant difference in the 3-month DASH score between the two groups (MD: –5.88, 95% CI: −9.72 to −2.03, *p* = 0.003), indicating that the PRP group demonstrated significantly superior functional improvement compared to the CS group at 3 months post-intervention. Significant heterogeneity was observed among the studies (Chi^2^ = 39.16, *p* < 0.00001, *I*^2^ = 90%). Subgroup analysis indicated that the gender distribution of patients was one source of the heterogeneity. Detailed information can be found in Supplementary file.

#### 3.3.6 DASH: 6 month

Three studies involving 170 patients reported the DASH score at 6 months (Figure 4C). The pooled analysis demonstrated a statistically significant difference in the 6-month DASH score between the two groups (MD: –14.42, 95% CI: −16.35 to −12.49, *p* < 0.00001), indicating that the PRP group demonstrated significantly superior functional improvement compared to the CS group at 6 months post-intervention. Furthermore, no heterogeneity was observed among the studies (Chi^2^ = 1.27, *p* = 0.53, *I*^2^ = 0%).

### 3.4 Secondary outcomes: ROM

#### 3.4.1 Abduction

**Figure 5:**
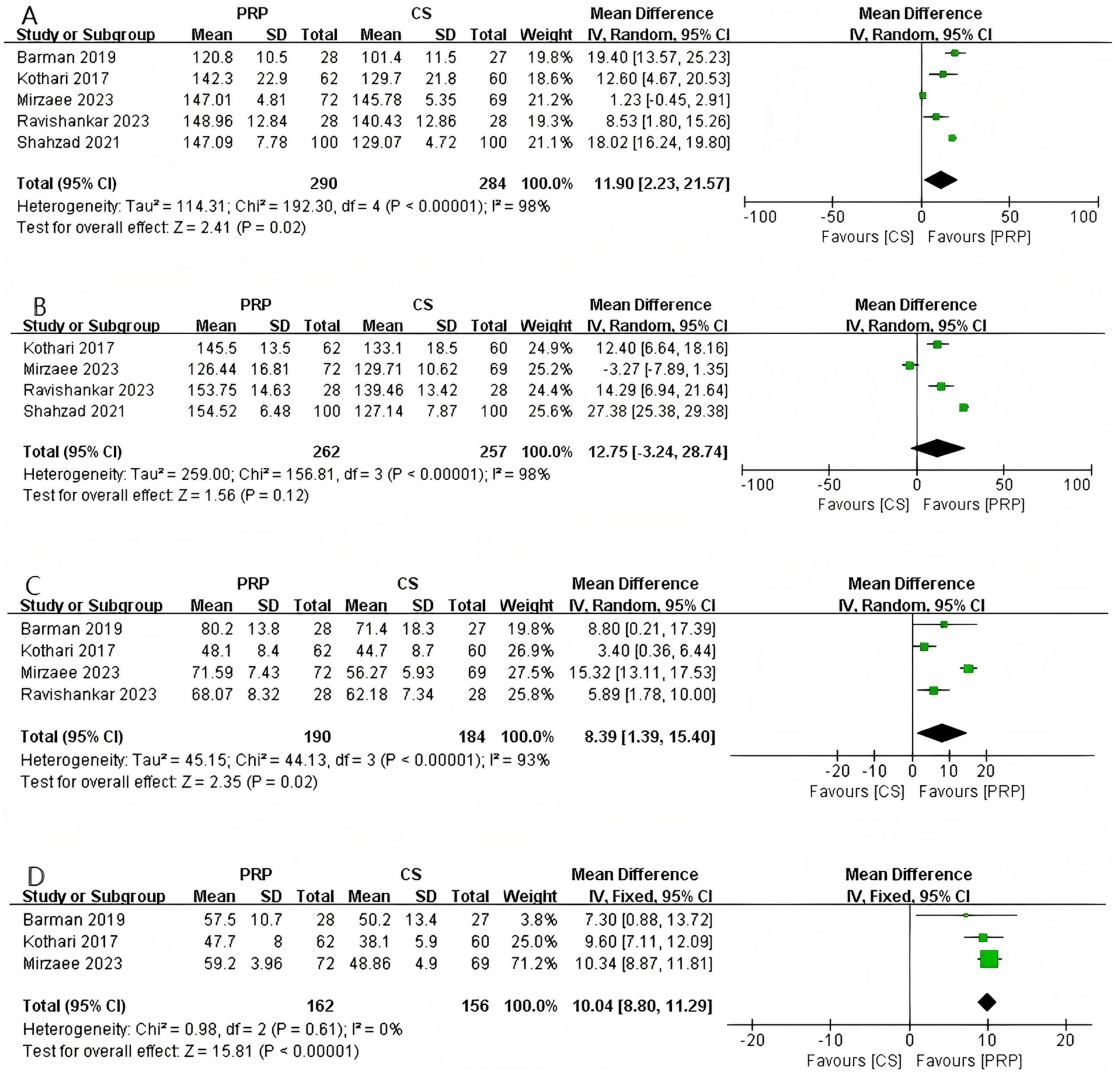
Forest plot of ROM: **(A)** Forest plot of abduction; **(B)** forest plot of flexion; **(C)** forest plot of external rotation; **(D)** forest plot of internal rotation.

As shown in Figure 5A, a total of 5 studies involving 574 patients reported shoulder abduction angle at the final follow-up. The pooled results demonstrated a statistically significant difference in abduction range of motion between the two groups (MD: 11.90, 95% CI: 2.23 to 21.57, *p* = 0.02), indicating that patients treated with PRP exhibited significantly greater shoulder abduction compared to those receiving CS injections. Significant heterogeneity was observed among the studies (Chi^2^ = 192.30, *p* < 0.00001, *I*^2^ = 98%). LOO analysis and subgroup analyses based on CS dosage and gender composition did not identify the source of heterogeneity.

#### 3.4.2 Flexion

Four studies involving 519 patients reported shoulder flexion angle at the final follow-up (Figure 5B). The pooled results indicated no significant difference in flexion range of motion between the two groups (MD: 12.75, 95% CI: −3.24 to 28.74, *p* = 0.12), suggesting that PRP and CS injections have comparable effects in improving shoulder flexion in patients with AC. Significant heterogeneity was observed among the studies (Chi^2^ = 156.81, *p* < 0.00001, *I*^2^ = 98%). Sensitivity analysis and subgroup analysis did not identify the source of heterogeneity. For further details, please refer to Supplementary file S2.

#### 3.4.3 External rotation

As shown in Figure 5C, a total of 4 studies involving 374 patients reported shoulder external rotation angle at the final follow-up. The pooled results demonstrated a significant difference between the two groups (MD: 8.39, 95% CI: 1.39 to 15.40, *p* = 0.02), indicating that PRP led to greater improvement in shoulder external rotation compared with CS in patients with AC. Although significant heterogeneity was observed among the studies (Chi^2^ = 44.13, *p* < 0.00001, *I*^2^ = 93%), sensitivity analysis suggested that the heterogeneity originated from the study by Shahzad et al. (18). After excluding this study, no heterogeneity remained (*I*^2^ = 0). For further details, please refer to Supplementary file S2.

#### 3.4.4 Internal rotation

Three studies (*n* = 318) reported shoulder internal rotation angle at the final follow-up (Figure 5D). The pooled results demonstrated a statistically significant difference between the two groups (MD: 10.04, 95% CI: 8.80 to 11.29, *p* < 0.00001), indicating that PRP was significantly superior to CS in improving shoulder internal rotation. No heterogeneity was observed among the studies (Chi^2^ = 0.98, *p* = 0.61, *I*^2^ = 0%).

### 3.5 Publication bias

According to the results of the Egger’s and Begg’s tests, the *p*-values for both tests were greater than 0.05 for all outcomes except the VAS at the 6-month follow-up, indicating a low likelihood of publication bias for these outcomes. However, for the 6-month VAS, the results of the two test methods were inconsistent: the Begg’s test did not indicate the presence of publication bias (*p* = 0.462), whereas the Egger’s test suggested a risk of bias (*p* = 0.006).

## 4 Discussion

This meta-analysis pooled data from 13 RCTs to comprehensively compare the efficacy of PRP versus CS injections for the treatment of AC. The main results indicated that although PRP and CS demonstrated comparable effects in terms of pain relief at 1 and 3 months, functional improvement at 1 month, and flexion, PRP exhibited significant advantages at the 6-month follow-up. Specifically, PRP was significantly superior to CS in pain control at 6 months, functional recovery from 3 to 6 months, and improvements in shoulder abduction, external rotation, and internal rotation range of motion.

Regarding the VAS score, the study by Yadav et al. (19) also found that PRP and CS exhibited comparable analgesic effects at the 1-month and 3-month follow-ups. However, the findings of Gupta et al. (20) and Kamra et al. (21) demonstrated that CS exhibited a significant advantage over PRP in terms of short-term pain relief. Early-stage pain in AC is primarily dominated by intense inflammatory pain, resulting from persistent stimulation of nociceptors by various inflammatory cytokines within the joint capsule, such as interleukin-6 (IL-6), tumor necrosis factor-alpha (TNF-α), and cyclooxygenase-1 (COX-1) (22). CS rapidly interrupt the inflammatory cascade by potently inhibiting COX and the production of pro-inflammatory cytokines such as IL-1β, IL-6, and TNF-α (23). This targeted mechanism allows CS to provide rapid pain relief in the early stages of AC. It is noteworthy, however, that PRP itself contains cytokines that antagonize inflammation (e.g., IL-1Ra) and promotes a microenvironment conducive to repair by modulating macrophage polarization from the pro-inflammatory M1 phenotype to the reparative M2 phenotype (24). This provides a basis for the early analgesic effect of PRP, explaining the similarity in short-term efficacy between the two treatments. Regarding the superior analgesic effect of PRP at the 6-month follow-up, this conclusion is supported by multiple studies (25–27). As AC progresses to the chronic phase, pain initially generated solely by inflammatory stimuli evolves into mixed pain: firstly, mechanical pain resulting from traction at the end range of motion due to fibrotic contracture of the joint capsule; and secondly, neuropathic pain caused by sensitization of local nerve fibers due to persistent chronic inflammation (28). At this stage, the limitations of CS injections become evident, as they cannot reverse established fibrosis and their efficacy diminishes over time. In contrast, the core advantage of PRP lies in its disease-modifying effects. Through the release of growth factors that promote fibroblast proliferation and organized collagen deposition (29), PRP aids in repairing the thickened and contracted joint capsule, restoring its extensibility and gliding function. As tissue structure improves, nociceptive pain triggered by end-range stretching and abnormal stimulation of mechanoreceptors will be fundamentally alleviated (30). Therefore, the analgesic superiority of PRP is essentially a natural outcome of its successful promotion of tissue regeneration and elimination of the root cause of pain. This characteristic of PRP is not an isolated phenomenon but aligns with evidence from its application in other chronic musculoskeletal disorders. A meta-analysis comparing PRP with CS for knee osteoarthritis (KOA) similarly indicated that while CS demonstrated superior analgesic effects in the short term (4–12 weeks), PRP exhibited significant and sustained advantages in long-term (6–12 months) pain relief and functional improvement (31). Although KOA and AC differ in pathology—the former primarily involves cartilage degeneration (32), while the latter is characterized by capsular fibrosis—both conditions share core processes of chronic inflammation and progressive structural deterioration. The “delayed yet sustained” efficacy pattern demonstrated by PRP in both disorders strongly suggests that its mechanism extends beyond mere anti-inflammatory effects and is more likely attributable to its disease-modifying potential. This provides a plausible biological explanation for its ability to maintain significant therapeutic effects even at the 6-month follow-up.

However, this meta-analysis identified significant heterogeneity among the studies included for the 1-month, 3-month, and 6-month VAS scores. Although sensitivity analysis did not identify the source of heterogeneity, subgroup analysis based on gender composition revealed distinctly different patterns of antinociceptive effects: in studies with a predominantly female population, CS demonstrated significantly superior antinociceptive efficacy compared to PRP at the 1-month follow-up; conversely, in studies with a predominantly male population, PRP showed a significant advantage as early as the 3-month follow-up. This finding is unlikely to be coincidental and strongly suggests the inherent sexual dimorphism in pain perception and analgesic mechanisms. Diffuse noxious inhibitory control (DNIC) is an endogenous “pain inhibits pain” analgesic mechanism. As females generally exhibit less efficient DNIC function than males, they tend to experience heightened pain sensitivity (33). Consequently, the potent and rapid-onset anti-inflammatory effects of CS may better address the urgent need for fast and powerful analgesia in female patients, thereby yielding superior therapeutic outcomes within the first month. In contrast, males, possessing a more robust endogenous analgesic baseline, may derive greater benefit from the reparative effects of PRP over time. Furthermore, subgroup analysis of the VAS at 3 months indicated that PRP demonstrated superior analgesic efficacy when the CS dosage was 1 mL, while the therapeutic effects of the two treatments were comparable when a 2 mL CS dosage was used. Although neither the sensitivity analysis nor the subgroup analysis definitively identified the source of heterogeneity in the 6-month VAS, both confirmed the reliability of the primary conclusion: PRP was significantly superior to CS in pain relief at 6 months post-intervention. In summary, we speculate that the high heterogeneity may stem from patient gender differences and the dosage of CS. Additionally, variations in injection techniques (such as injection depth and the use of ultrasound guidance) could also be potential sources of heterogeneity. Regarding the assessment of publication bias, the results of Egger’s test and Begg’s test for the 6-month VAS were inconsistent: Egger’s test suggested the possible presence of publication bias (*p* = 0.006), whereas Begg’s test showed no statistical significance (*p* = 0.462). This discrepancy may be attributed to the greater sensitivity of Egger’s test to heterogeneity in effect sizes and to studies with small sample sizes, while Begg’s test might have had insufficient statistical power under the present data distribution. Nevertheless, given the positive signal indicated by Egger’s test, caution is warranted when interpreting the pooled results for the 6-month VAS.

Regarding functional recovery and range of motion improvement, this meta-analysis indicates that PRP treatment offers significant advantages over CS, particularly evident at the 3-month and 6-month follow-ups. The findings of multiple randomized controlled trials are consistent with the conclusions of this study (20, 21, 34). No significant difference in functional improvement was observed between the two treatments at the 1-month mark, which may be attributed to the rapid analgesic effect achieved by CS through its anti-inflammatory action, thereby contributing to a certain degree of functional recovery in the short term. In contrast, the growth factors contained within PRP support the entire process of wound healing and repair (13). Although this biological process develops relatively slowly, it establishes a structural foundation for functional recovery at 3 and 6 months, which is also consistent with the significant improvements observed in the DASH scores at these time points. In terms of the ROM, the marked superiority of PRP in abduction, external rotation, and internal rotation further corroborates its role in modulating pathological processes such as capsular fibrosis and contracture. Abduction and rotational movements of the shoulder are highly dependent on the extensibility of the glenohumeral joint capsule and the smooth gliding of the humeral head within the glenoid fossa. Specific capsuloligamentous structures, such as the middle glenohumeral ligament and the inferior glenohumeral ligament complex, play key roles in restricting excessive translation of the humeral head, thereby directly influencing the terminal angles of abduction and rotation (35, 36). PRP directly improved mechanical kinematic performance in these directions by promoting tissue healing and collagen reorganization. Notably, no significant difference in flexion range of motion was observed between the two groups. This may be attributed to the greater reliance of shoulder flexion on compensatory mechanisms of the scapulothoracic joint, rather than solely on the extent of capsular release in the glenohumeral joint. Using three-dimensional motion analysis, McClure et al. (37) quantified the scapular contribution to shoulder flexion in healthy individuals, demonstrating that the scapulothoracic joint accounts for approximately one-third of the total range of motion during arm elevation. Furthermore, Lin et al. (38) investigated the impact of shoulder stiffness on kinematics and observed that subjects with stiff shoulders exhibited altered scapular movement patterns and rhythm in an effort to maintain functional upper limb mobility. These findings directly support the notion that shoulder flexion relies more heavily on compensatory mechanisms compared with other planes of motion.

Notably, although no significant heterogeneity was observed in the DASH scores at 1 month and 6 months, the 3-month DASH results exhibited substantial heterogeneity (*I*^2^ = 90%). LOO analysis did not identify the source of heterogeneity but confirmed the robustness of this finding. Further subgroup analysis revealed that the heterogeneity primarily stemmed from the demographic characteristics of the included study populations, specifically the gender distribution of the patients. No heterogeneity was observed among studies with a higher proportion of male patients (*I*^2^=0%), and a significant difference was detected between PRP and CS treatment (*p* < 0.00001). A similar absence of heterogeneity (*I*^2^ = 0%) and a significant intergroup difference (*p* = 0.0004) were also found in studies with a higher proportion of female patients. It is noteworthy that in studies with a higher proportion of male patients, the PRP group demonstrated superior functional recovery at the 3-month post-treatment follow-up. This finding may be related to the microenvironment of PRP-mediated tissue healing. Testosterone has been shown to stimulate the proliferation of human dermal fibroblasts and enhance their migratory capacity via an androgen receptor-dependent pathway (39). Furthermore, relevant molecular biology studies indicate that during tendon healing, the androgen receptor signaling pathway is activated in the early healing phase and regulates the expression of a series of genes associated with cell proliferation and matrix synthesis (such as type II myofibers genes encoding type II myosin heavy chain and parvalbumin) (40). The higher androgen levels in males may provide a more synergistic microenvironment for the growth factors in PRP, thereby amplifying its reparative efficacy. In contrast, estrogen exerts a complex bidirectional regulatory effect on the fibrotic process (41, 42). It may inhibit the excessive activation of fibroblasts at specific stages, which, while beneficial for reducing pathological scar formation, could also potentially slow the pace of PRP-mediated structural remodeling of the joint capsule to some extent. The above discussion on sex differences explains why PRP demonstrated superior outcomes in studies with a higher proportion of male participants. Regarding the clinical outcomes of ROM, significant heterogeneity was observed among studies for all movements except internal rotation (*I*^2^ = 0%). Sensitivity analysis identified the study by Mirzaee et al. (43) as the source of heterogeneity. After its exclusion, heterogeneity was eliminated, and PRP still showed a significant advantage (*p* = 0.0001, *I*^2^ = 0%). It was found that patient population characteristics could be a source of heterogeneity. Additionally, differences in rehabilitation protocols following treatment might also contribute to heterogeneity among studies. Sensitivity and subgroup analyses did not identify the source of heterogeneity for abduction and flexion.

Significant heterogeneity was observed in the analyses of VAS and some ROM measurements. To explore its sources, we systematically performed sensitivity analysis (LOO) and subgroup analyses (based on gender and corticosteroid dose). The results indicated that gender was a profound moderating factor: CS injections demonstrated superior short-term analgesic effects in studies with predominantly female participants, whereas PRP showed earlier advantages in studies with predominantly male participants. Although most of the heterogeneity could not be fully explained by a single factor (potentially due to insufficiently reported details such as PRP preparation protocols and injection techniques), all analyses confirmed the robustness of the core conclusion: PRP provided sustained superior outcomes compared to CS at the 6-month follow-up in terms of pain relief, functional improvement, and ROM. In accordance with the Cochrane Handbook, a random-effects model was employed to provide conservative estimates. We recommend that future studies standardize the reporting of intervention details to further elucidate the sources of heterogeneity.

## 5 Limitation

This meta-analysis has several limitations: (1) Heterogeneity was observed in the source study data for some outcome measures. In accordance with the Cochrane Handbook, a random-effects model was applied to address this heterogeneity; however, heterogeneity introduced by factors such as variations in PRP preparation protocols, injection techniques, and post-treatment rehabilitation plans could not be disregarded. (2) The longest follow-up time point in this analysis was defined as 6 months, which may be insufficient to evaluate the true long-term efficacy for a chronic condition like AC. Future RCTs with longer follow-up durations are warranted. (3) Although outcomes from the included studies were reported independently, certain assessment variables (e.g., range of motion and functional scores) may be intrinsically correlated. By analyzing them separately, the results could have been skewed.

## 6 Conclusion

Based on the results of this meta-analysis, a novel “stage-adaptive” treatment strategy for AC is proposed: CS is recommended as a high-efficacy intervention in the acute phase, while PRP is suggested as the recommended treatment plan for the chronic/fibrotic stage. The core of this strategic shift lies in moving from merely controlling symptoms toward altering the natural course of the disease, representing an evolution in the AC treatment paradigm. Furthermore, an innovative finding of this study is the confirmation that sex is a significant moderator of treatment response. The pathological mechanisms of AC may exhibit sexual dimorphism, and understanding the role of sex factors opens new avenues for precision medicine in AC. Future research should further explore biomarkers such as sex hormones and genetic background to establish individualized treatment prediction models. Clinical decision-making should comprehensively evaluate the patient’s disease stage, primary symptoms, and expectations regarding long-term prognosis.

## Data Availability

The original contributions presented in the study are included in the article/Supplementary material, further inquiries can be directed to the corresponding author.

## Supplementary material

The Supplementary material for this article can befound online at: https://www.frontiersin.org/articles/10.3389/fmed.2026.1766836/full#supplementary-material
