# Supplementary material for: Comparison of the efficacy of platelet-rich plasma versus corticosteroid in the treatment of adhesive capsulitis: a systematic review and meta-analysis based on randomized controlled trials
Source: Front Med (Lausanne). 2026 Feb 5;13:1766836. doi: 10.3389/fmed.2026.1766836 (PMC12916625; doi:10.3389/fmed.2026.1766836)
Supplement: Supplementary file 1 [file Table_1.docx]

****Pubmed 32****

****#1: Platelet-Rich Plasma[MeSH Terms]****

****#2: ((Plasma, Platelet-Rich[Title/Abstract]) OR (Platelet Rich Plasma[Title/Abstract])) OR (PRP[Title/Abstract])****

****#3: #1 OR #2****

****#4: Adrenal Cortex Hormones[MeSH Terms]****

****#5: ((((((((Hormones, Adrenal Cortex[Title/Abstract]) OR (Corticoid[Title/Abstract])) OR (Adrenal Cortex Hormone[Title/Abstract])) OR (Cortex Hormone, Adrenal[Title/Abstract])) OR (Hormone, Adrenal Cortex[Title/Abstract])) OR (Corticosteroid[Title/Abstract])) OR (Corticoids[Title/Abstract])) OR (Corticosteroids[Title/Abstract])) OR (Glucocorticoids[Title/Abstract])****

****#6: #4 OR #5****

****#7: Bursitis[MeSH Terms]****

****#8: (((((((((((((((Bursitides[Title/Abstract]) OR (Adhesive Capsulitis[Title/Abstract])) OR (Adhesive Capsulitides[Title/Abstract])) OR (Capsulitides, Adhesive[Title/Abstract])) OR (Capsulitis, Adhesive[Title/Abstract])) OR (Frozen Shoulder[Title/Abstract])) OR (Frozen Shoulders[Title/Abstract])) OR (Shoulder, Frozen[Title/Abstract])) OR (Adhesive Capsulitis of the Shoulder[Title/Abstract])) OR (Shoulder Adhesive Capsulitis[Title/Abstract])) OR (Adhesive Capsulitides, Shoulder[Title/Abstract])) OR (Adhesive Capsulitis, Shoulder[Title/Abstract])) OR (Capsulitides, Shoulder Adhesive[Title/Abstract])) OR (Capsulitis, Shoulder Adhesive[Title/Abstract])) OR (Shoulder Adhesive Capsulitides[Title/Abstract])) OR (Capsulitis[Title/Abstract])****

****#9: #7 OR #8****

****#10: #3 AND #6 AND #9****

****WOS 65****

****#1: TS=(Platelet-Rich Plasma)****

****#2: TS=(Plasma, Platelet-Rich) OR TS=(Platelet Rich Plasma) OR TS=(PRP)****

****#3: #1 OR #2****

****#4: TS=(Adrenal Cortex Hormones)****

****#5: TS=(Hormones, Adrenal Cortex) OR TS=(Corticoid) OR TS=(Adrenal Cortex Hormone) OR TS=(Cortex Hormone, Adrenal) OR TS=(Hormone, Adrenal Cortex) OR TS=(Corticosteroid) OR TS=(Corticoids) OR TS=(Corticosteroids) OR TS=(Glucocorticoids)****

****#6: #4 OR #5****

****#7:** TS=(Bursitis)**

****#8: TS=(Bursitides) OR TS=(Adhesive Capsulitis) OR TS=(Adhesive Capsulitides) OR TS=(Capsulitides, Adhesive) OR TS=(Capsulitis, Adhesive) OR TS=(Frozen Shoulder) OR TS=(Frozen Shoulders) OR TS=(Shoulder, Frozen) OR TS=(Adhesive Capsulitis of the Shoulder) OR TS=(Shoulder Adhesive Capsulitis) OR TS=(Adhesive Capsulitides, Shoulder) OR TS=(Adhesive Capsulitis, Shoulder) OR TS=(Capsulitides, Shoulder Adhesive) OR TS=(Capsulitis, Shoulder Adhesive) OR TS=(Shoulder Adhesive Capsulitides) OR TS=(Capsulitis[Title/Abstract])****

****#9: #7 OR #8****

****#10: #3 AND #6 AND #9****

****Embase 49****

****#1: 'thrombocyte rich plasma'/exp****

****#2: 'platelet rich plasma':ab,ti OR 'platelet-rich plasma':ab,ti OR 'thrombocyte rich plasma':ab,ti****

****#3: #1 OR #2****

****#4:**** 'corticosteroid'/exp

****#5: 'adrenal cortex hormone':ab,ti OR 'adrenal cortex hormones':ab,ti OR 'adrenal cortical hormone':ab,ti OR 'adrenal cortical hormones':ab,ti OR 'adrenal cortical steroid':ab,ti OR 'adrenal steroid':ab,ti OR 'adrenal steroid hormone':ab,ti OR 'adreno cortical steroid':ab,ti OR 'adreno corticosteroid':ab,ti OR 'adrenocortical hormone':ab,ti OR 'adrenocortical steroid':ab,ti OR adrenocorticosteroid:ab,ti OR 'cortical steroid':ab,ti OR corticosteroid:ab,ti OR corticoid:ab,ti****

****#6: #4 OR #5****

****#7: 'humeroscapular periarthritis'/exp****

****#8: 'adhesive capsulitis':ab,ti OR 'adhesive capsulitis, shoulder':ab,ti OR 'adhesive shoulder capsulitis':ab,ti OR 'calcifying periarthritis, shoulder':ab,ti OR 'calcifying shoulder periarthritis':ab,ti OR 'humeroscapular arthritis':ab,ti OR 'painful stiff shoulder':ab,ti OR 'periarthritis humeri scapularis':ab,ti OR 'periarthritis humero scapularis':ab,ti OR 'periarthritis humeroscapularis':ab,ti OR 'periarthritis scapulohumeralis':ab,ti OR 'peritendinitis humeroscapularis':ab,ti OR 'periarthropathia humeroscapularis':ab,ti OR 'phs syndrome':ab,ti OR 'scapulo humeral periarthritis':ab,ti OR 'scapulohumeral periarthritis':ab,ti OR 'shoulder capsulitis, adhesive':ab,ti OR 'shoulder periarthritis':ab,ti OR 'shoulder periarthritis, calcifying':ab,ti OR 'stiff shoulder, painful':ab,ti OR 'tenosynovitus humeroscapularis':ab,ti OR 'humeroscapular periarthritis':ab,ti****

****#9: #7 OR #8****

****#10: #3 AND #6 AND #9****

**Cochrane Library **25****

****#1:**** [Platelet-Rich Plasma] explode all trees

****#2: (Plasma, Platelet-Rich):ti,ab,kw OR (Platelet Rich Plasma):ti,ab,kw OR (PRP):ti,ab,kw****

****#3: #1 OR #2****

****#4:**** [Adrenal Cortex Hormones] explode all trees

****#5: (Hormones, Adrenal Cortex):ti,ab,kw OR (Corticoid):ti,ab,kw OR (Adrenal Cortex Hormone):ti,ab,kw OR (Cortex Hormone, Adrenal):ti,ab,kw OR (Hormone, Adrenal Cortex):ti,ab,kw OR (Corticosteroid):ti,ab,kw OR (Corticoids):ti,ab,kw OR (Corticosteroids):ti,ab,kw OR (Glucocorticoids):ti,ab,kw****

****#6: #4 OR #5****

****#7:**** [**Bursitis**] explode all trees

****#8: (Bursitides):ti,ab,kw OR (Adhesive Capsulitis):ti,ab,kw OR (Adhesive Capsulitides):ti,ab,kw OR (Capsulitides, Adhesive):ti,ab,kw OR (Capsulitis, Adhesive):ti,ab,kw OR (Frozen Shoulder):ti,ab,kw OR (Frozen Shoulders):ti,ab,kw OR (Shoulder, Frozen):ti,ab,kw OR (Adhesive Capsulitis of the Shoulder):ti,ab,kw OR (Shoulder Adhesive Capsulitis):ti,ab,kw OR (Adhesive Capsulitides, Shoulder):ti,ab,kw OR (Adhesive Capsulitis, Shoulder):ti,ab,kw OR (Capsulitides, Shoulder Adhesive):ti,ab,kw OR (Capsulitis, Shoulder Adhesive):ti,ab,kw OR (Shoulder Adhesive Capsulitides):ti,ab,kw OR (Capsulitis)****

****#9: #7 OR #8****

****#10: #3 AND #6 AND #9****
