# Supplementary material for: Comparison of the efficacy of platelet-rich plasma versus corticosteroid in the treatment of adhesive capsulitis: a systematic review and meta-analysis based on randomized controlled trials
Source: Front Med (Lausanne). 2026 Feb 5;13:1766836. doi: 10.3389/fmed.2026.1766836 (PMC12916625; doi:10.3389/fmed.2026.1766836)
Supplement: Supplementary file 2 [file Table_2.docx]

**Sensitivity Analysis:** “leave one out” method (LOO)

1. The LOO method for 1-month VAS. (B)The LOO for 3-month VAS. (C)The LOO for 6-month VAS. (D)The LOO for 3-month DASH. (E)The LOO for abduction. (F)The LOO for flexion. (G)The LOO for external rotation.

**A**


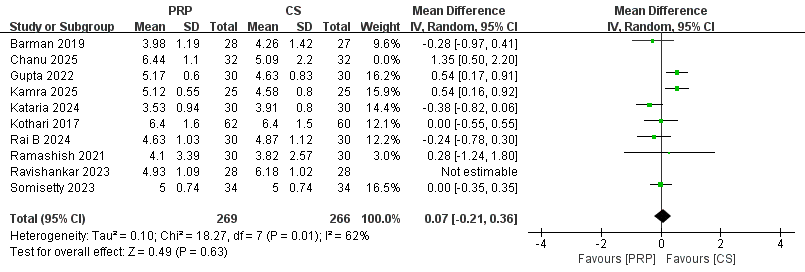


**B**

**
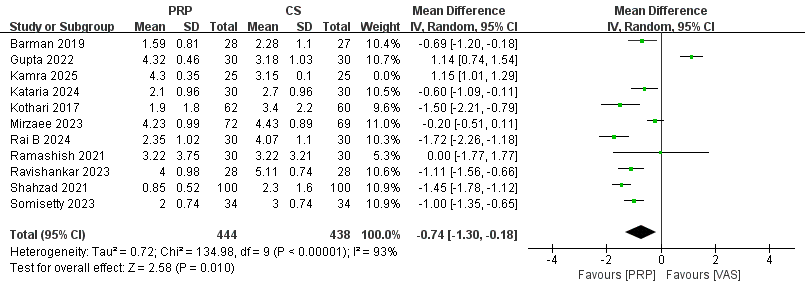
**

**C**

**
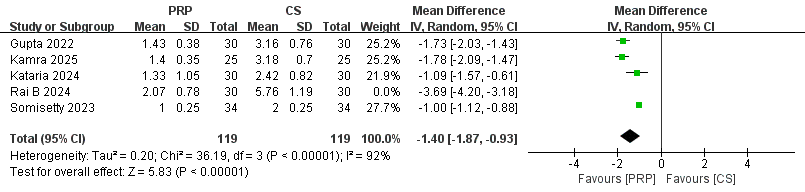
**

**D**

**
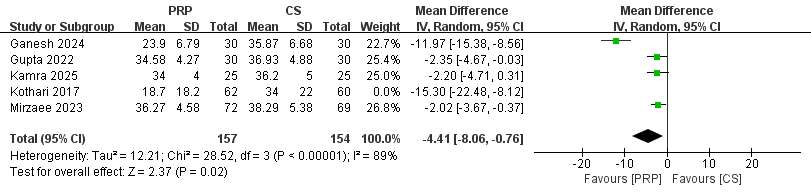
**

**E**

**
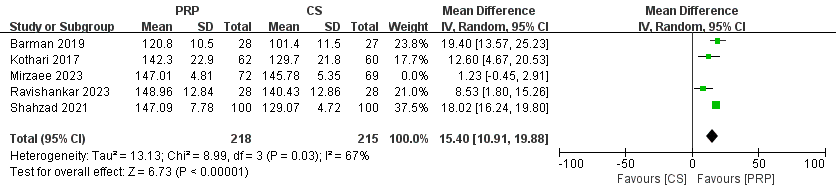
**

**F**

**
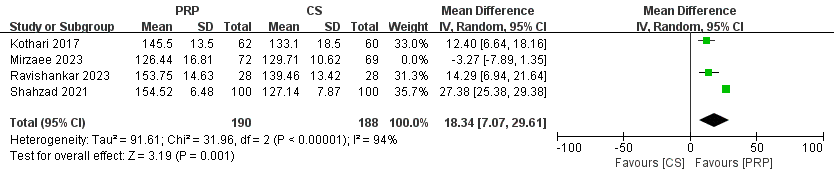
**

**G**

**
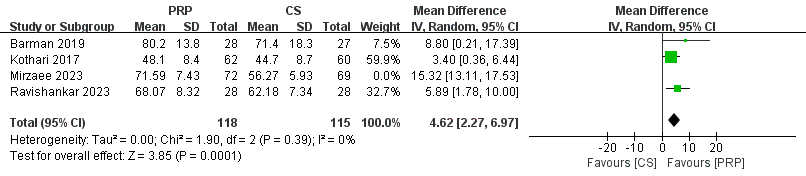
**
