# Supplementary material for: Comparison of the efficacy of platelet-rich plasma versus corticosteroid in the treatment of adhesive capsulitis: a systematic review and meta-analysis based on randomized controlled trials
Source: Front Med (Lausanne). 2026 Feb 5;13:1766836. doi: 10.3389/fmed.2026.1766836 (PMC12916625; doi:10.3389/fmed.2026.1766836)
Supplement: Supplementary file 3 [file Table_3.docx]

**Subgroup analysis：**

1.Subgroup analysis of 1-month VAS (A-Steroid dosage, B-Gender)

**A**

**
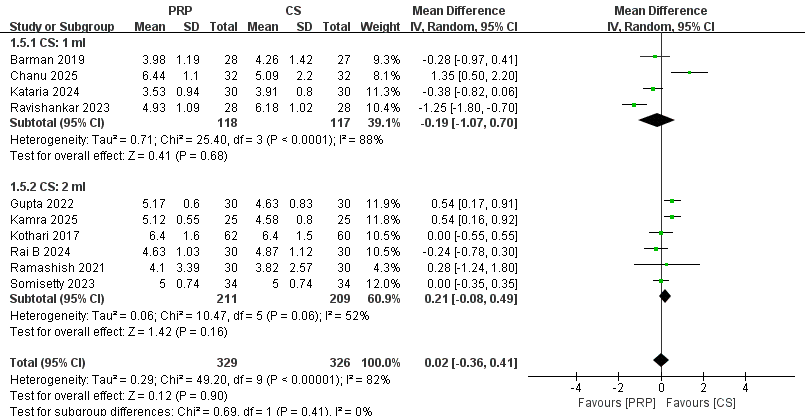
**

**B**

**
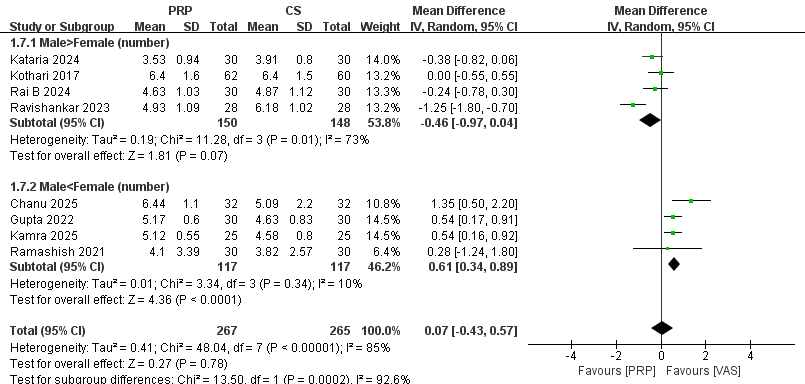
**

2.Subgroup analysis of 3-month VAS (A-Steroid dosage, B-Gender)

**A**

**
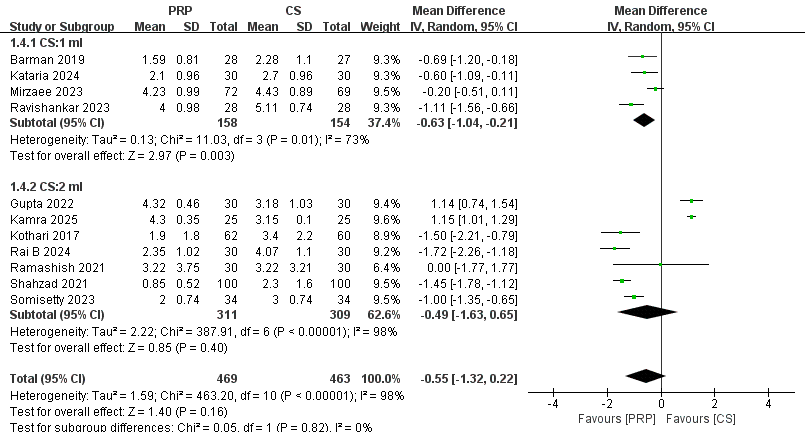
**

**B**

**
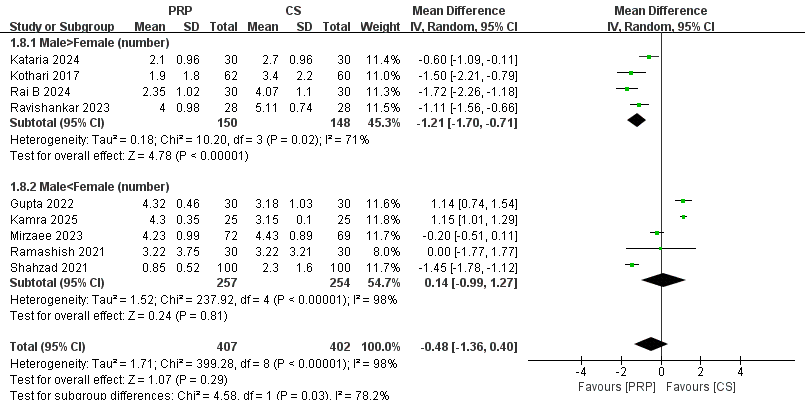
**

3.Subgroup analysis of 6-month VAS (A-Gender)

**A**

**
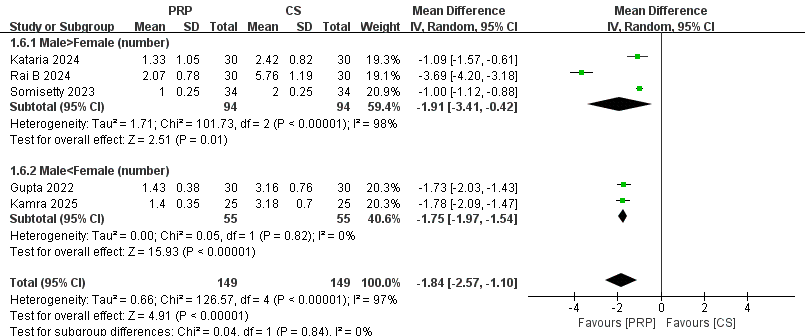
**

4.Subgroup analysis of 3-month DASH (A-Steroid dosage, B-Gender)

**A**

**
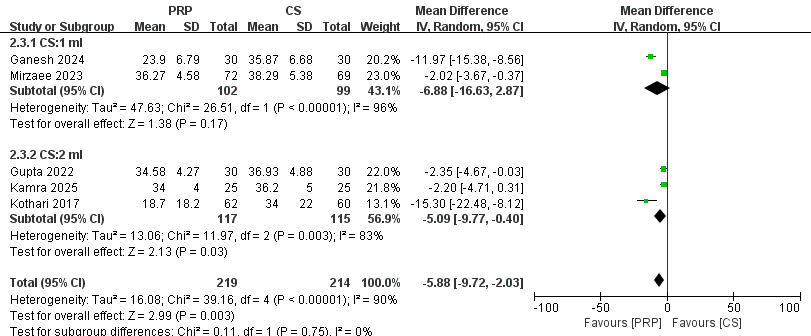
**

**B**


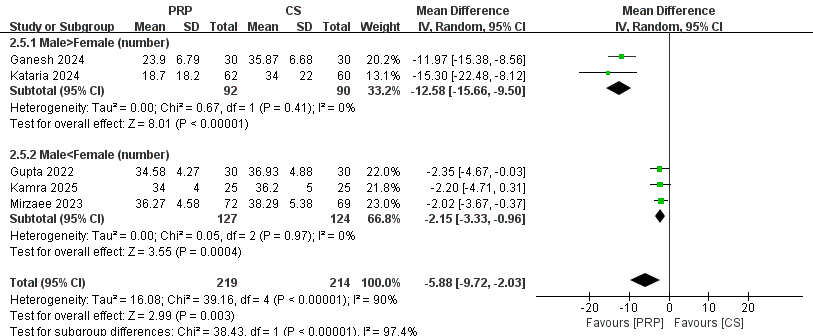


5.Subgroup analysis of abduction (A-Steroid dosage, B-Gender)

**A**

**
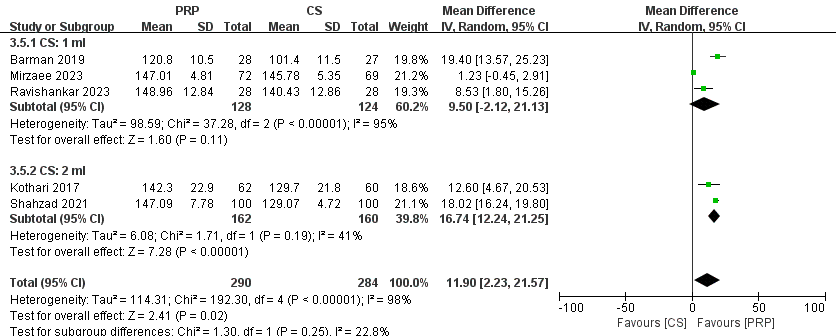
**

**B**

**
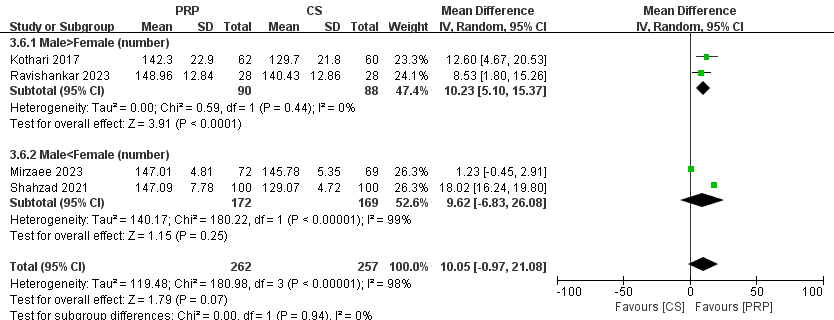
**

5.Subgroup analysis of flexion (A-Steroid dosage, B-Gender)

**A**


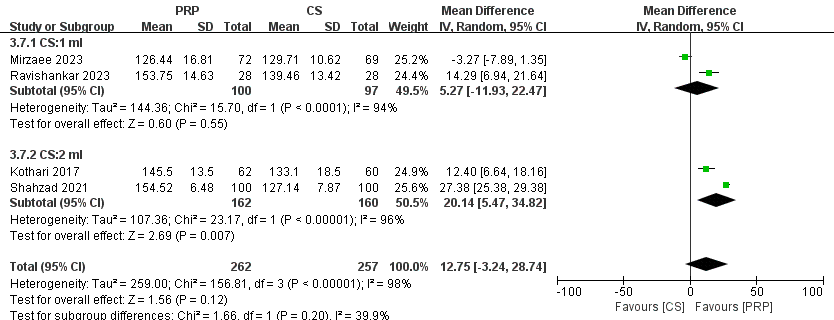


**B**

**
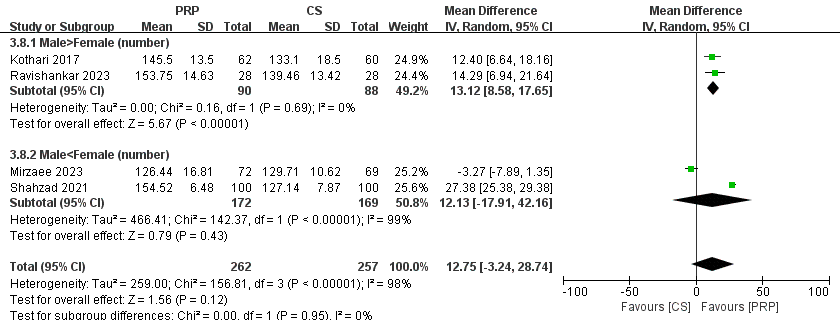
**
