# Supplementary material for: Comparison of the efficacy of platelet-rich plasma versus corticosteroid in the treatment of adhesive capsulitis: a systematic review and meta-analysis based on randomized controlled trials
Source: Front Med (Lausanne). 2026 Feb 5;13:1766836. doi: 10.3389/fmed.2026.1766836 (PMC12916625; doi:10.3389/fmed.2026.1766836)

**Figure1:** Funnel plots of the primary outcomes

A: Funnel plot of the 1-month VAS; B: Funnel plot of the 3-month VAS; C: Funnel plot of the 6-month VAS; D: Funnel plot of the 1-month DASH; E: Funnel plot of the 3-month DASH; F: Funnel plot of the 6-month DASH.

**A**


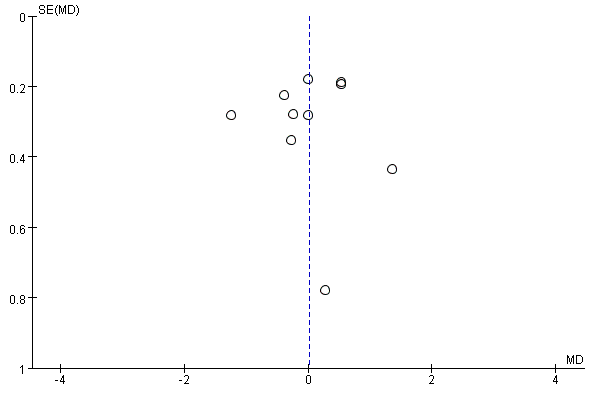


**B**


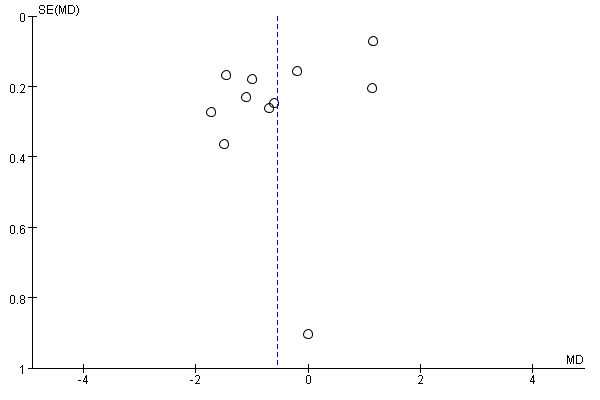


**C**


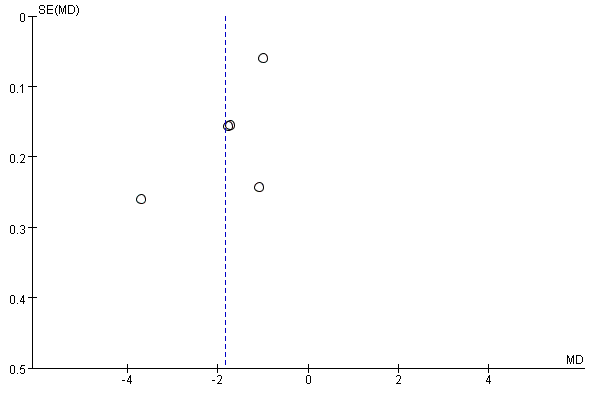


**D**


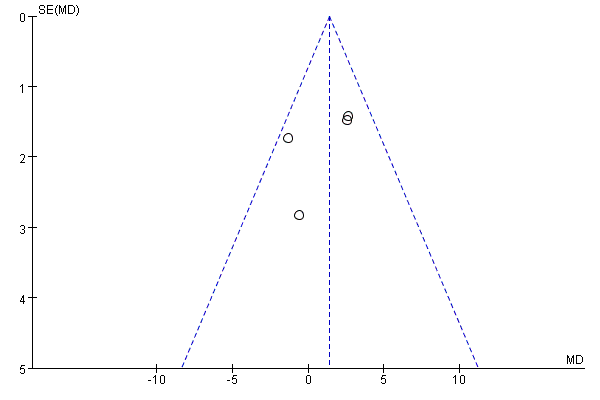


**E**


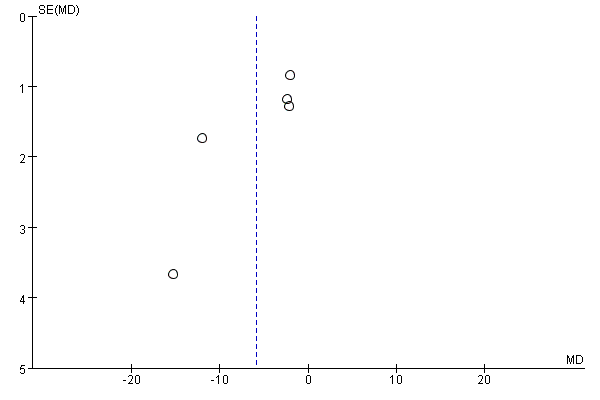


**F**


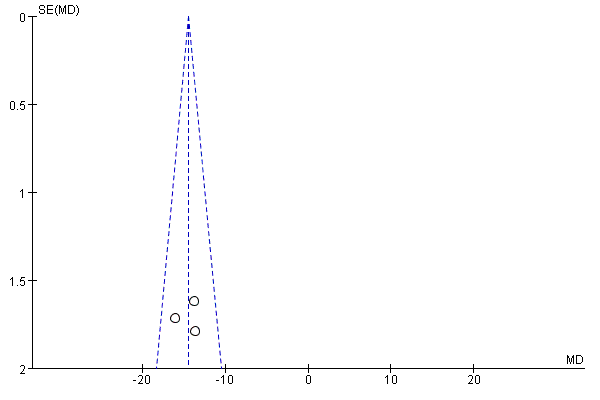


**Figure2:** Funnel plots of the secondary outcomes

A: Funnel plot of the abduction; B: Funnel plot of the flexion; C: Funnel plot of the external rotation; D: Funnel plot of the internal rotation.

**A**


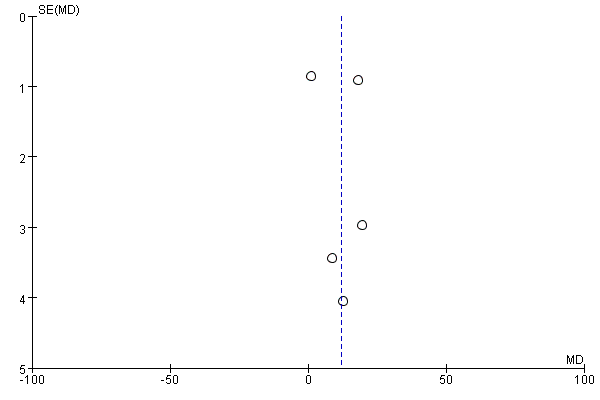


**B**


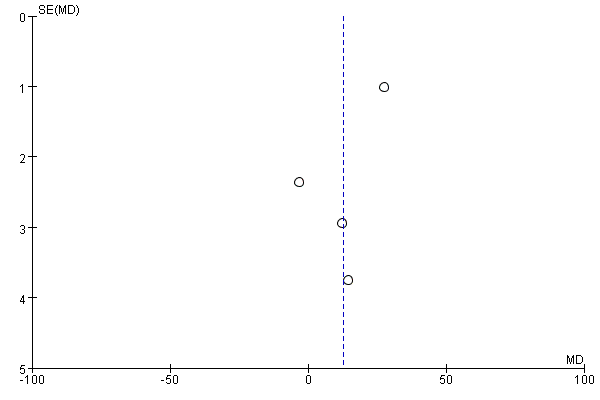


**C**


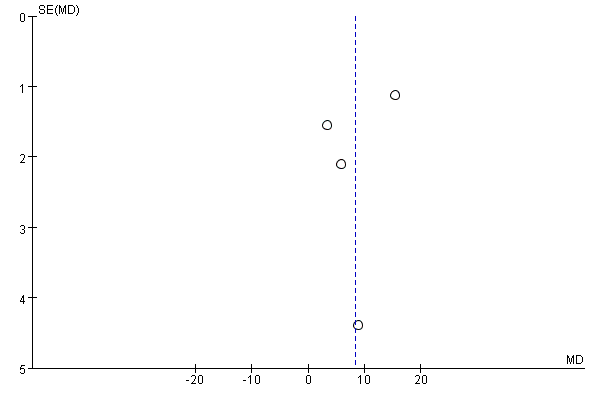
 **D**


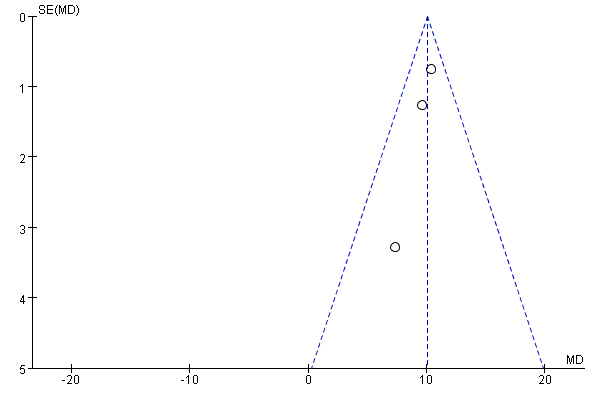

Supplement: Supplementary file 4 [file Table_4.docx]
