# Supplementary material for: Comparison of the efficacy of platelet-rich plasma versus corticosteroid in the treatment of adhesive capsulitis: a systematic review and meta-analysis based on randomized controlled trials
Source: Front Med (Lausanne). 2026 Feb 5;13:1766836. doi: 10.3389/fmed.2026.1766836 (PMC12916625; doi:10.3389/fmed.2026.1766836)

Egger's tests of the outcomes：

A: 1-month VAS; B: 3-month VAS; C: 6-month VAS; D: 1-month DASH; E: 3-month DASH; F: 6-month DASH; G: Abduction; H: Flexion; I: External rotation; J: Internal rotation.

**A**

**
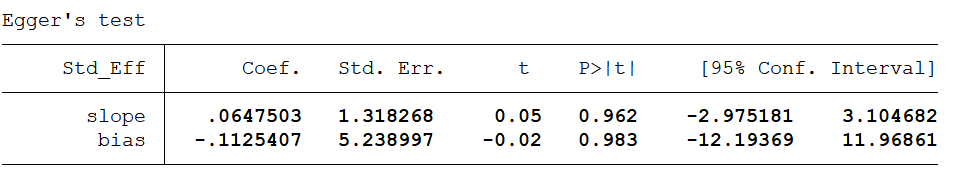
**


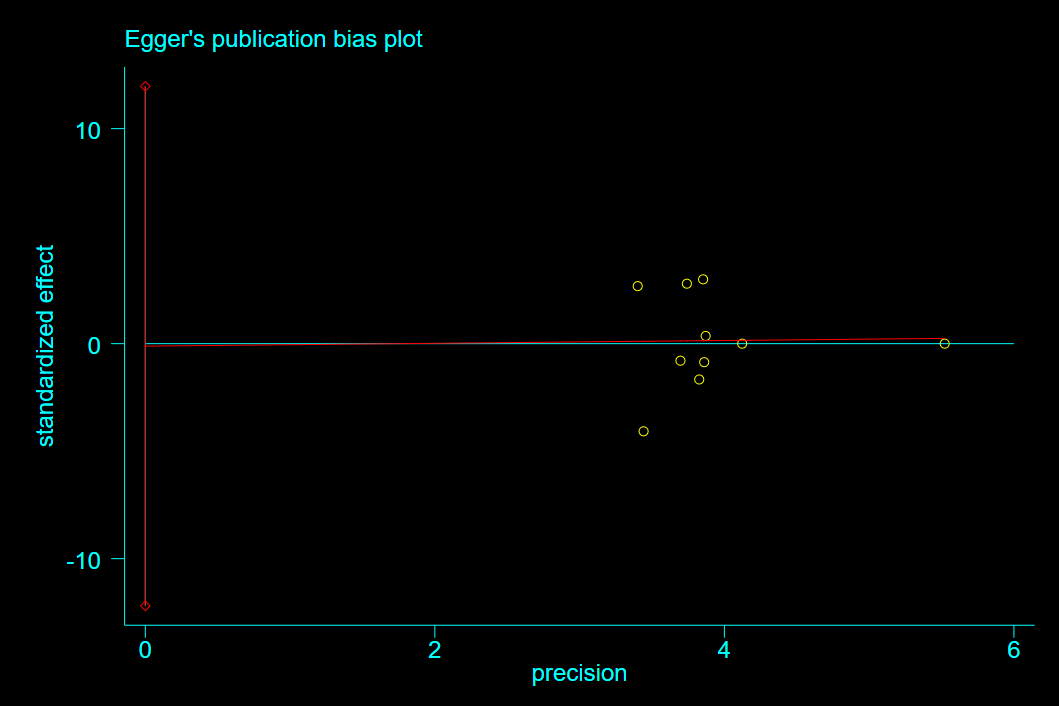


**B**

**
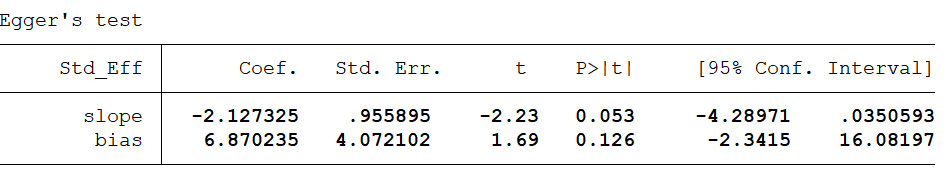
**


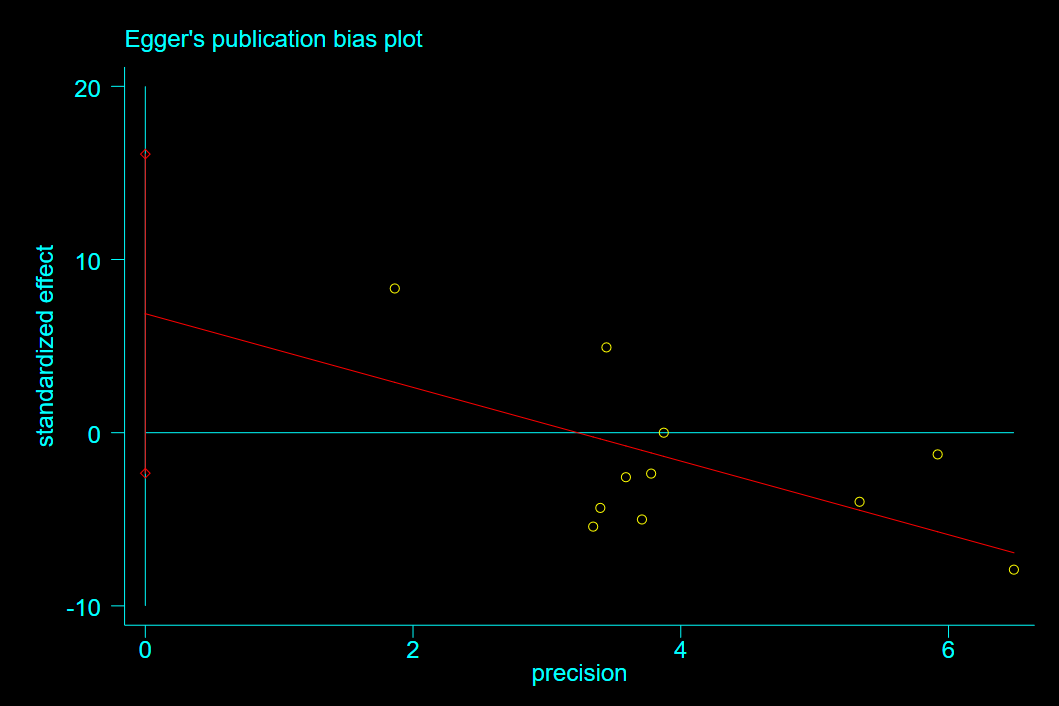


**C**

**
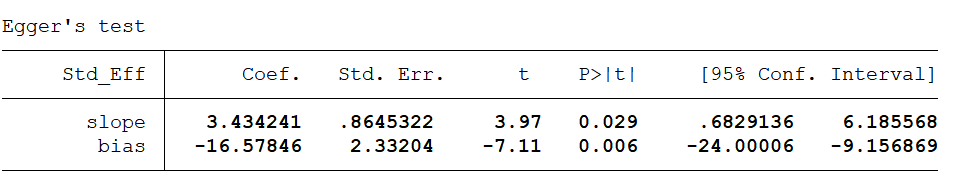
**


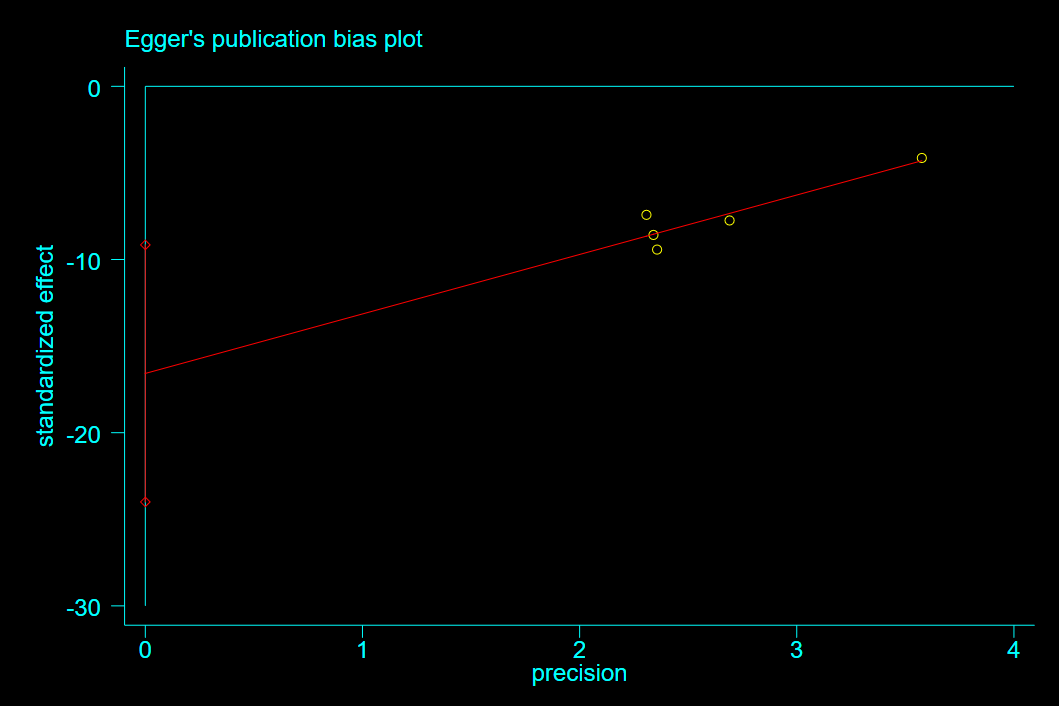


**D**

**
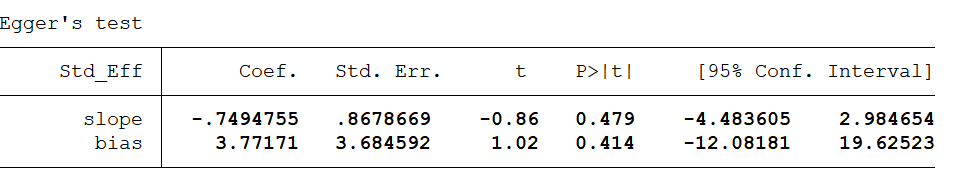
**


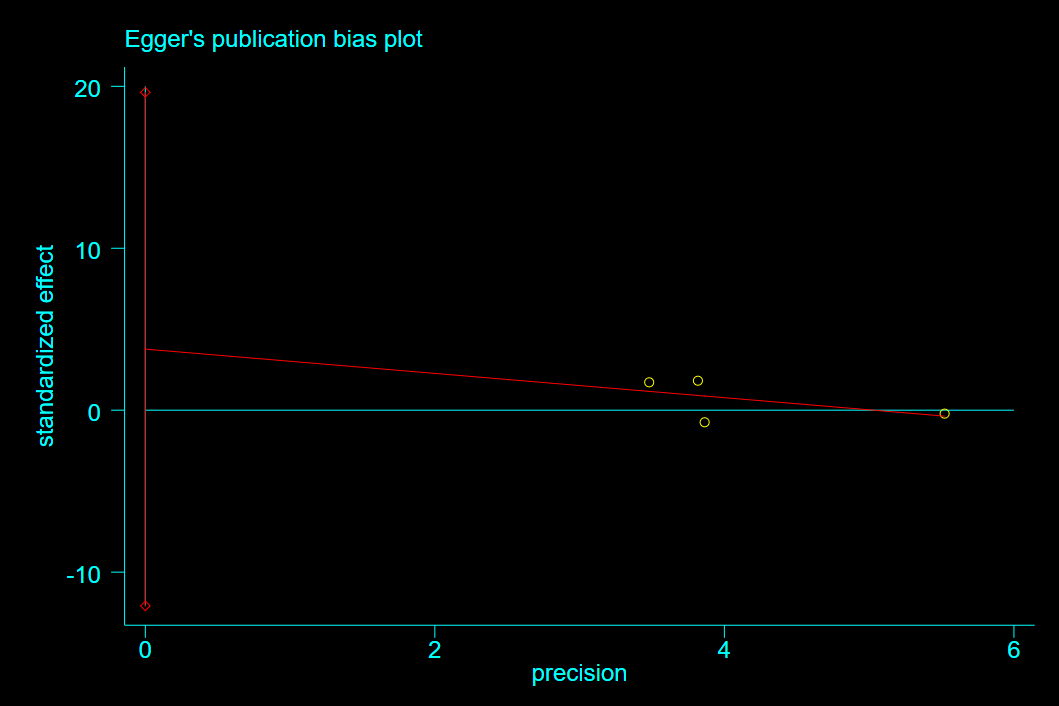


**E**

**
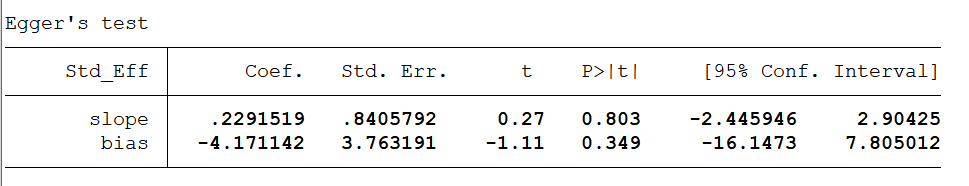
**


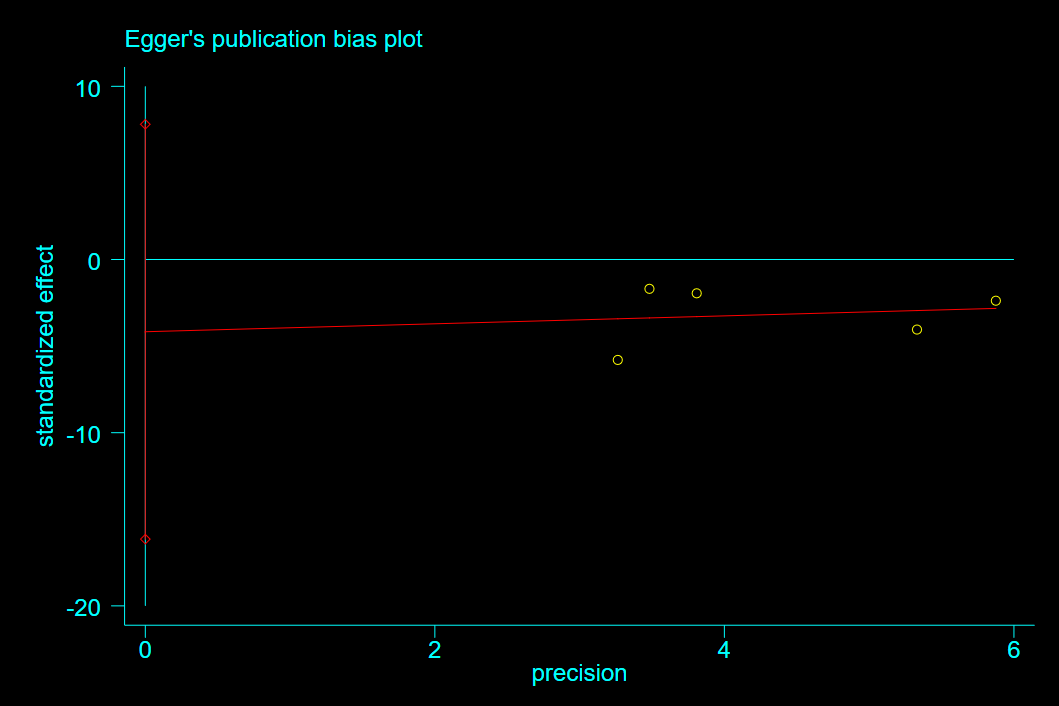


**F**

**
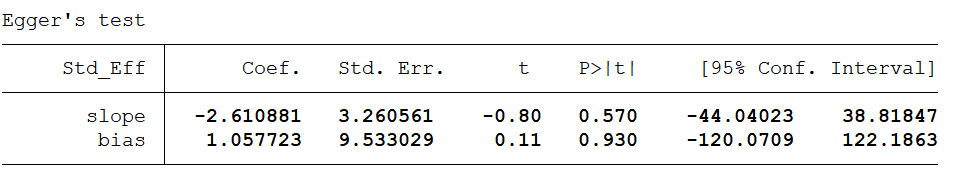
**


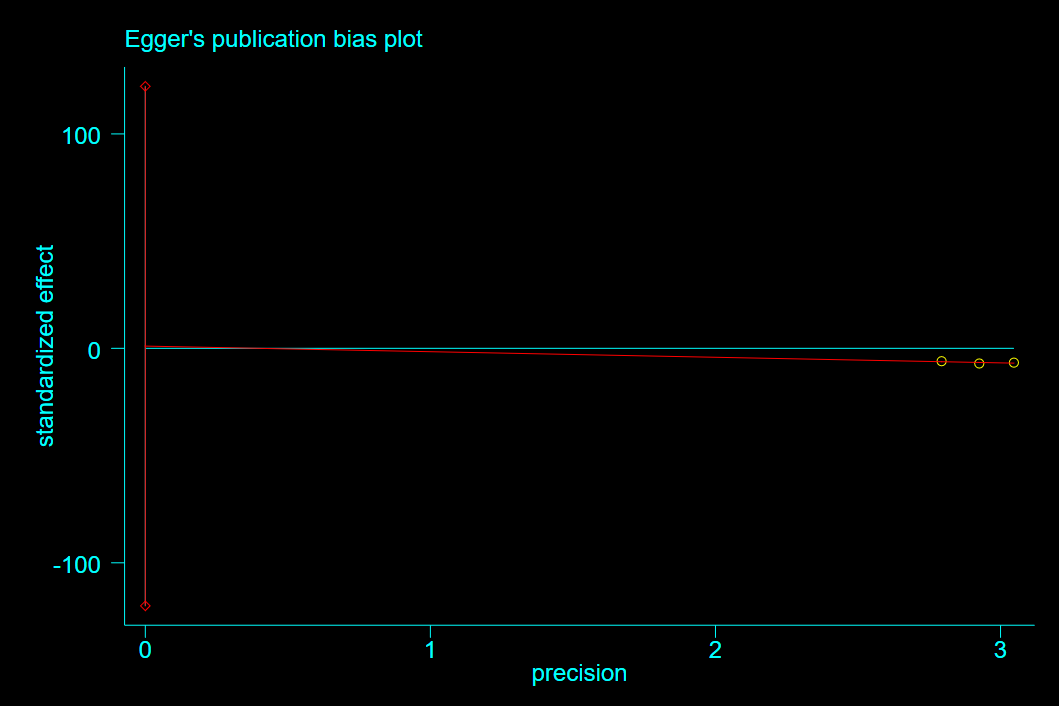


**G**

**
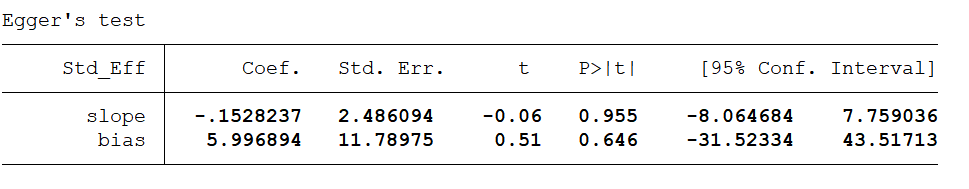
**


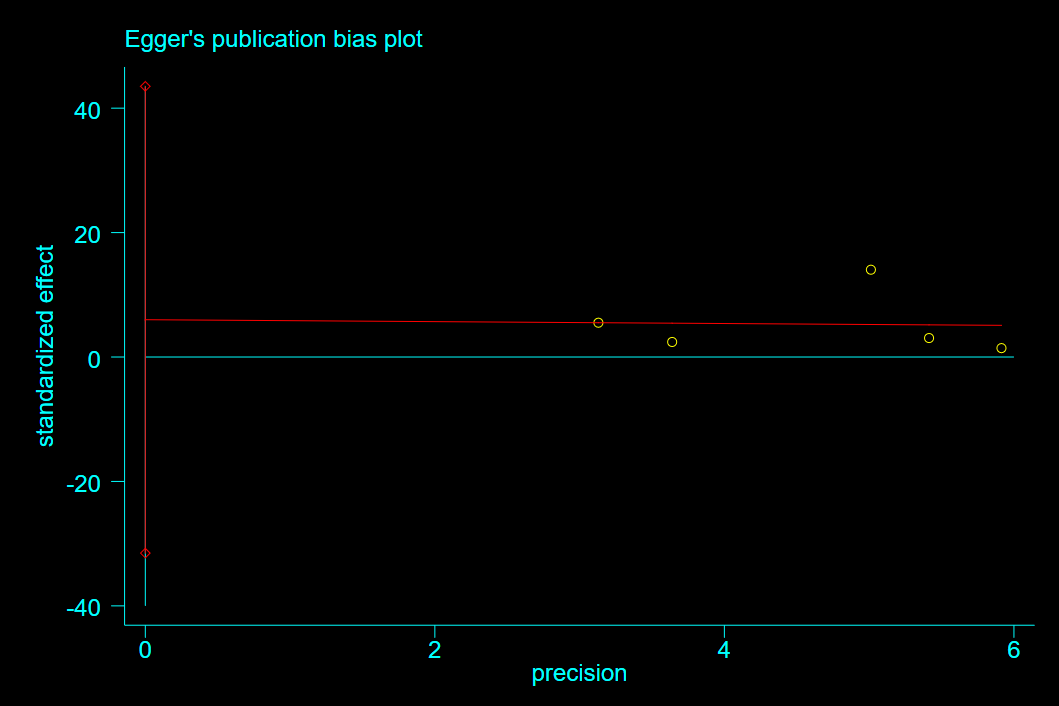


**H**

**
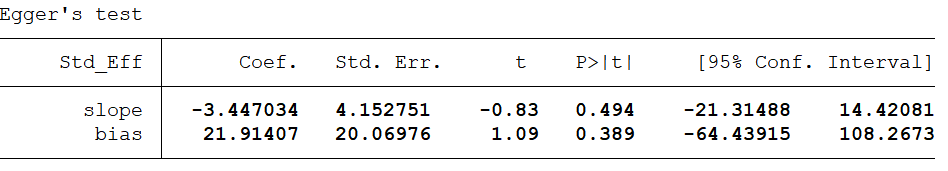
**


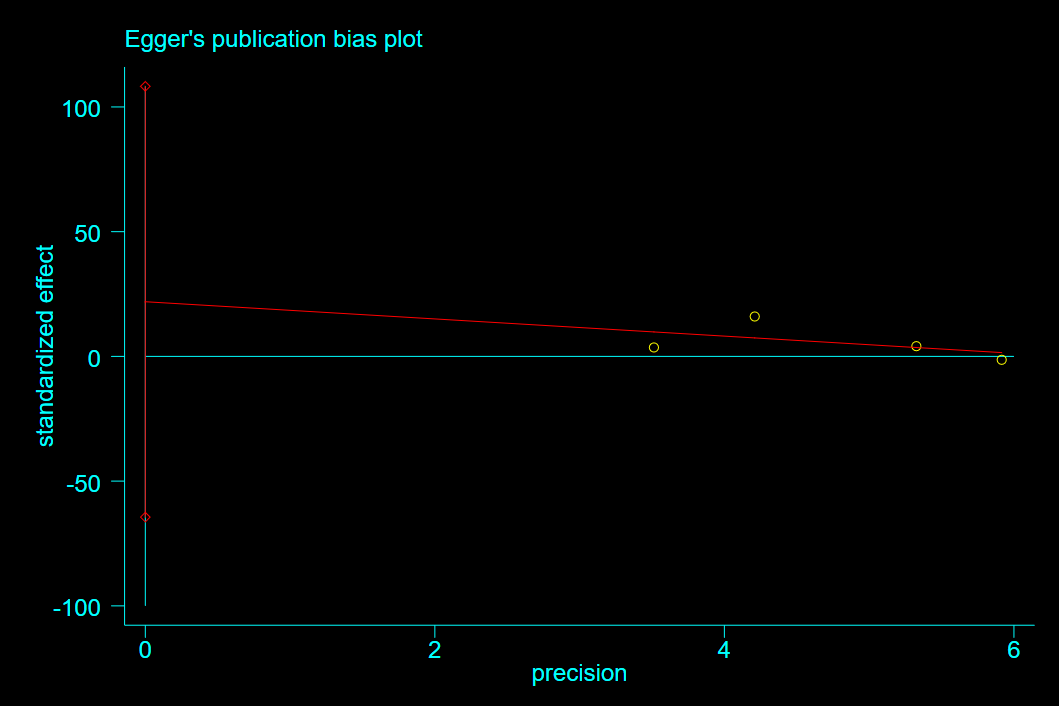


**I**

**
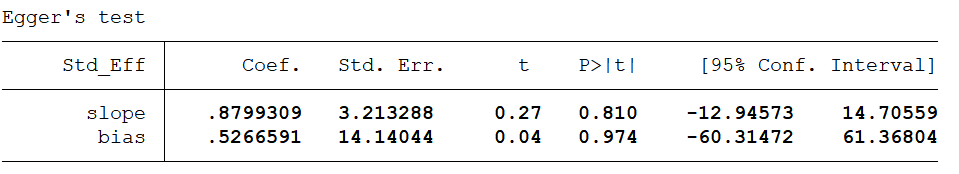
**


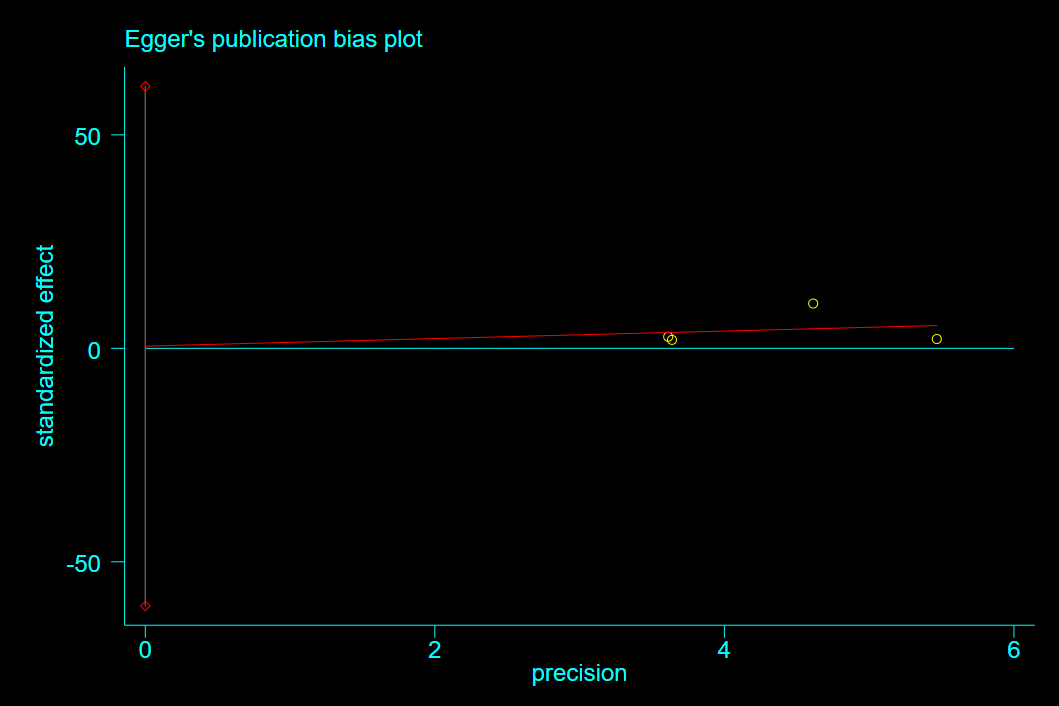


**J**

**
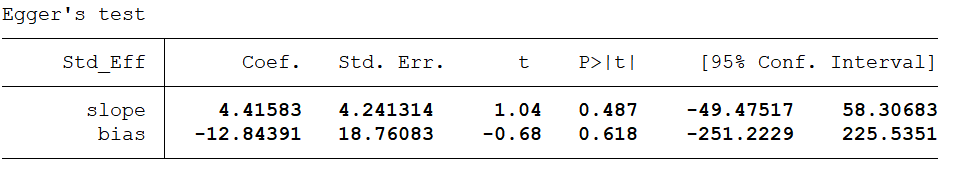
**


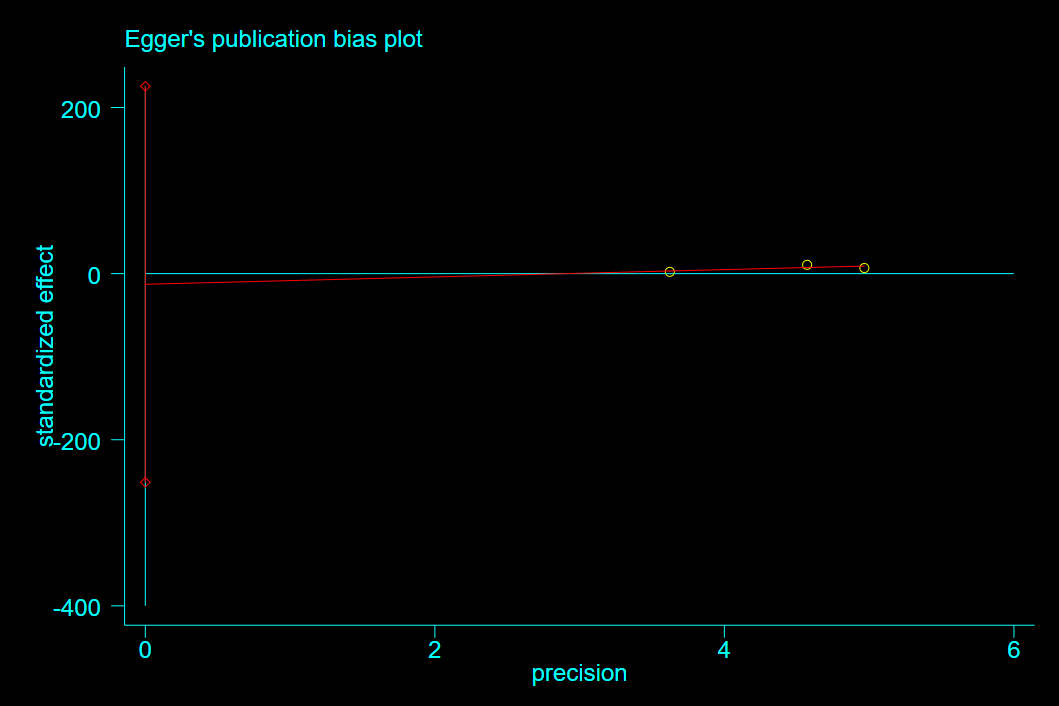

Supplement: Supplementary file 5 [file Table_5.docx]
