# Supplementary material for: Comparison of the efficacy of platelet-rich plasma versus corticosteroid in the treatment of adhesive capsulitis: a systematic review and meta-analysis based on randomized controlled trials
Source: Front Med (Lausanne). 2026 Feb 5;13:1766836. doi: 10.3389/fmed.2026.1766836 (PMC12916625; doi:10.3389/fmed.2026.1766836)

Begg's tests of the outcomes：

**Begg's tests of the outcomes**

| Primary outcomes | P value | Secondary outcomes | P value |
| --- | --- | --- | --- |
| 1-month VAS | 0.858 | Abduction | 0.221 |
| 3-month VAS | 0.755 | Flexion | 0.308 |
| 6-month VAS | 0.462 | External rotation | 0.734 |
| 1-month DASH | 0.734 | Internal rotation | 1.000 |
| 3-month DASH | 0.462 |  |  |
| 6-month DASH | 1.000 |  |  |

**Figures:**

A: 1-month VAS; B: 3-month VAS; C: 6-month VAS; D: 1-month DASH; E: 3-month DASH; F: 6-month DASH; G: Abduction; H: Flexion; I: External rotation; J: Internal rotation.

A


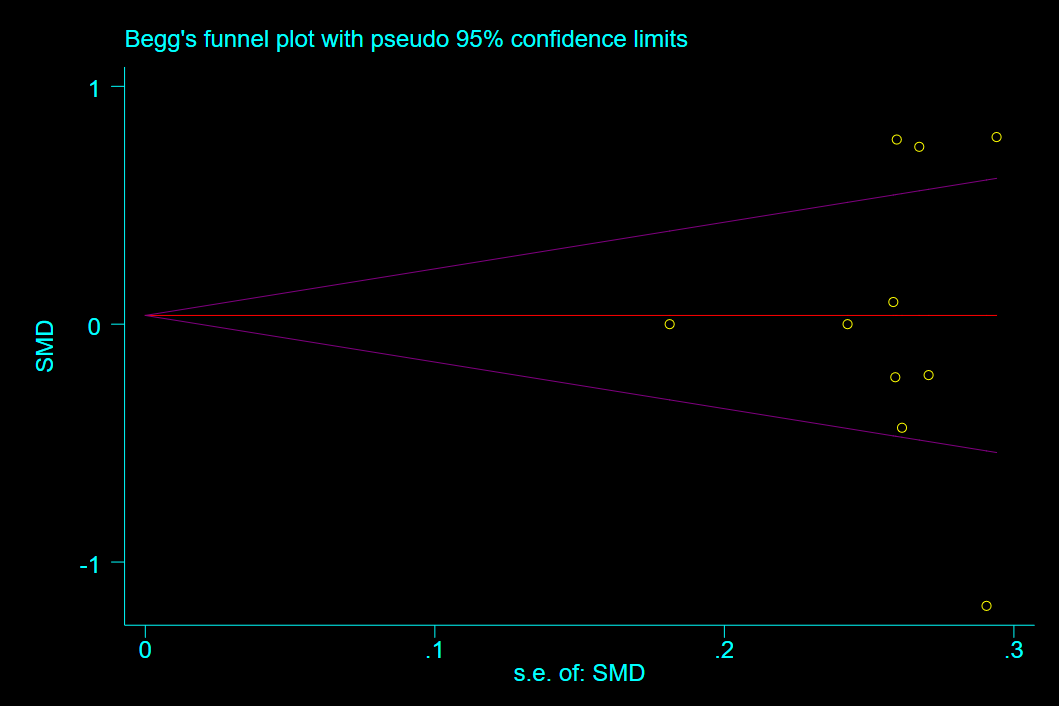


B


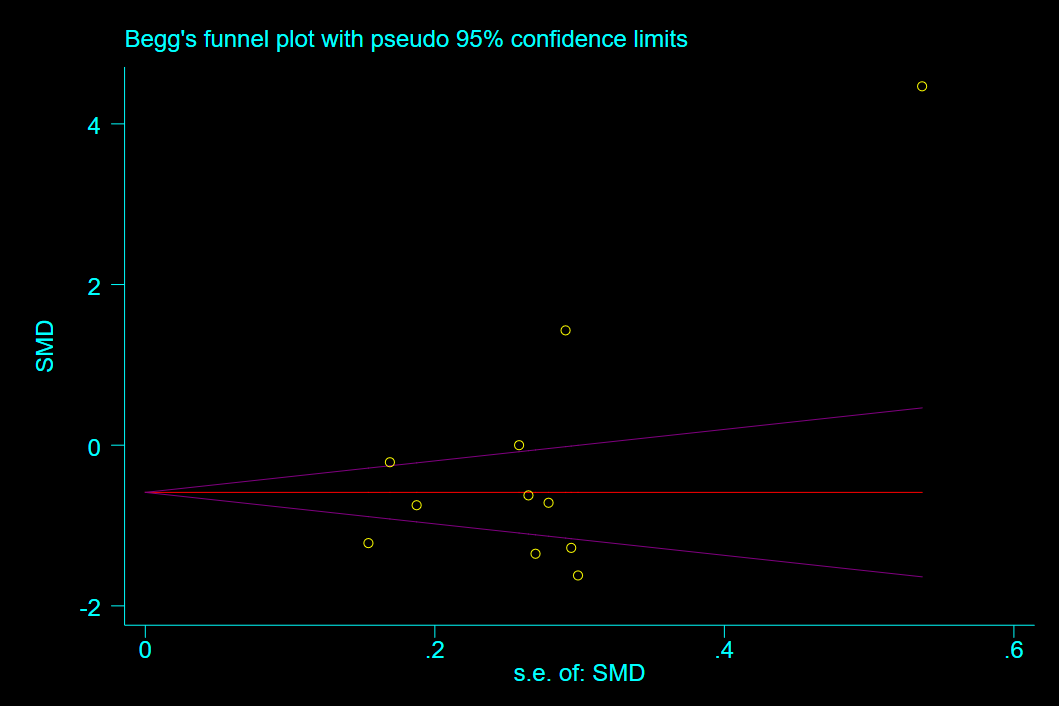


C


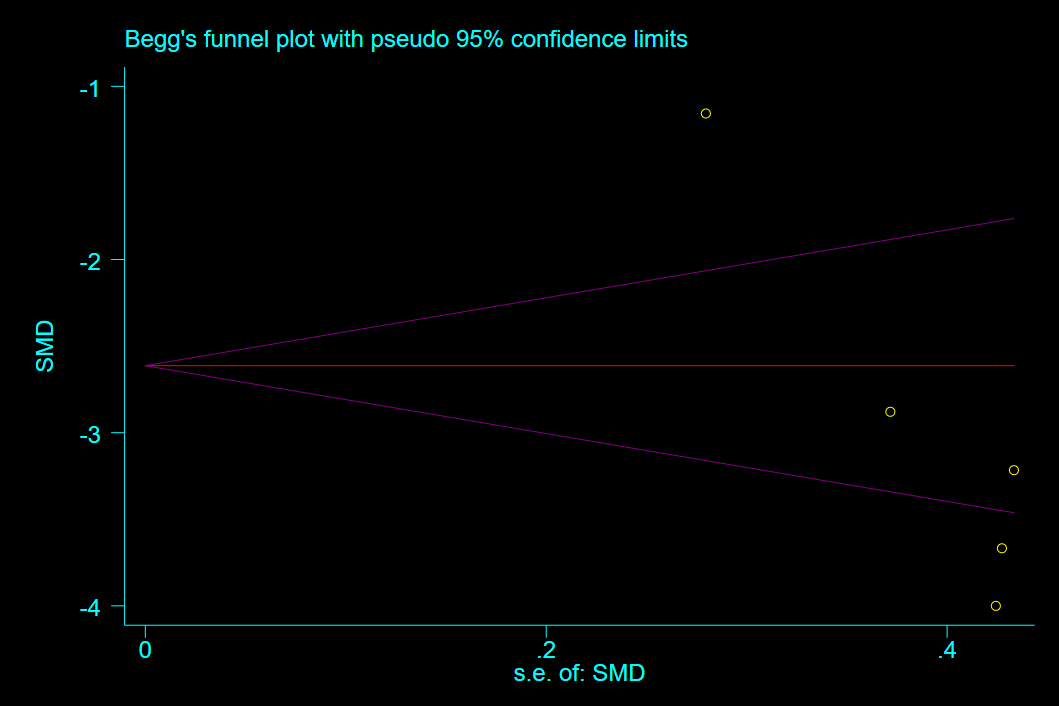


D


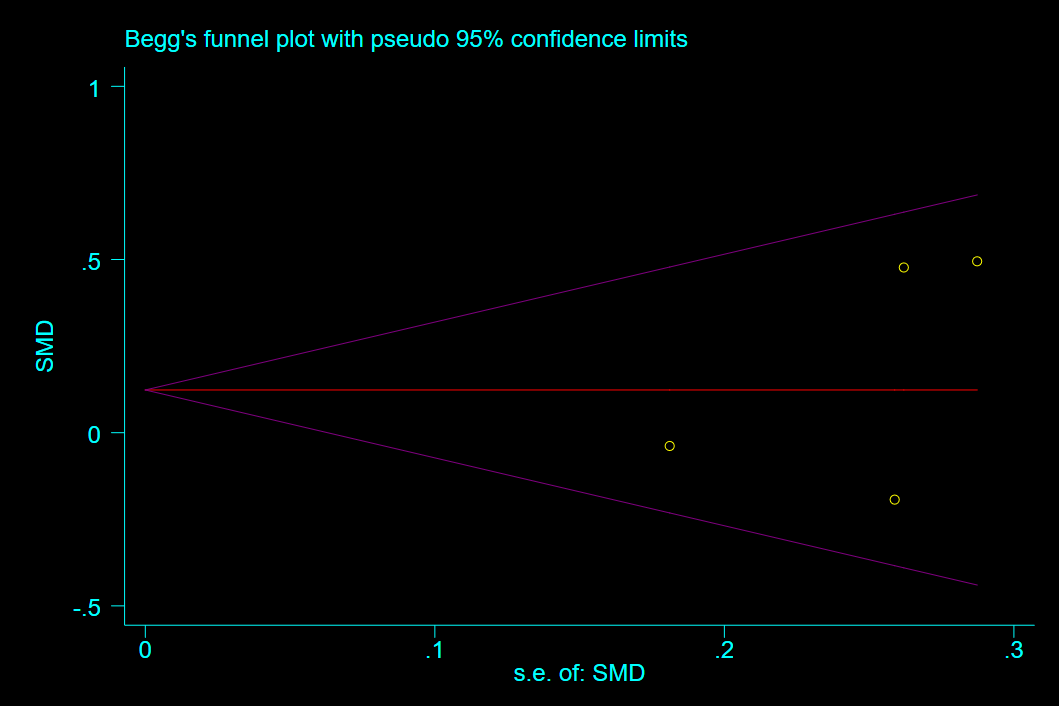


E


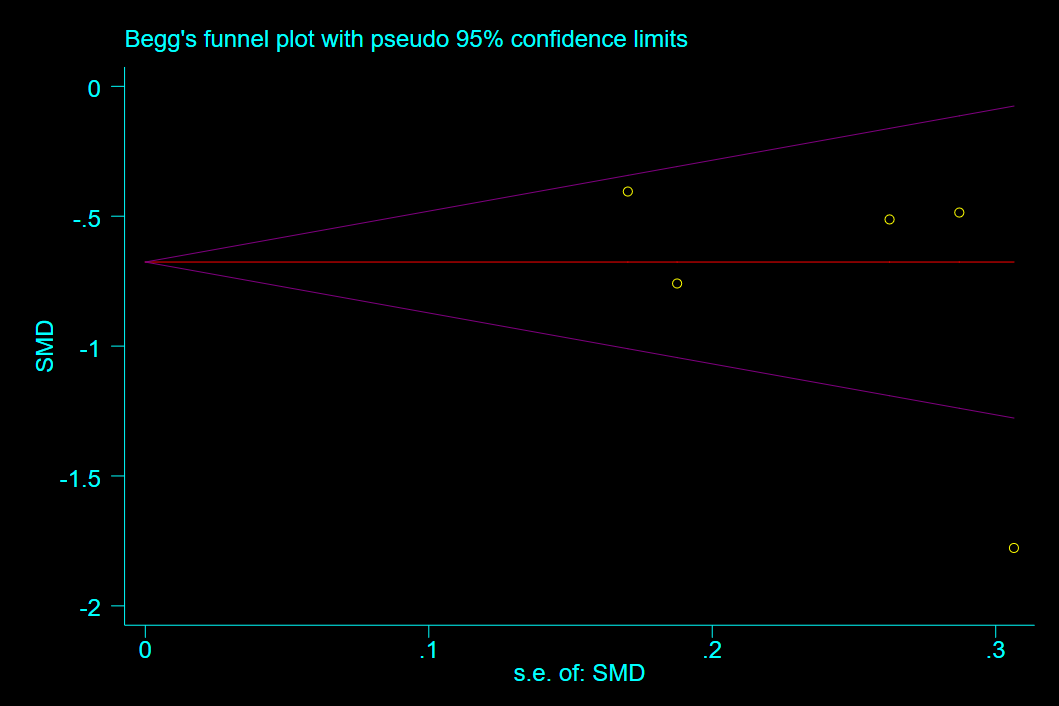


F


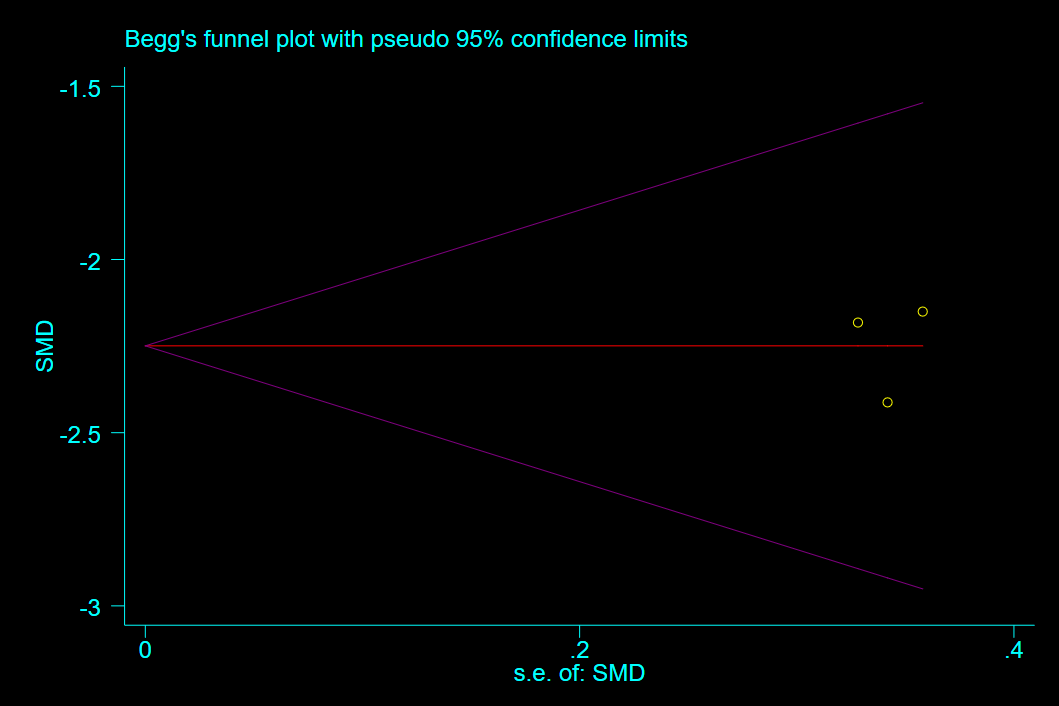


G


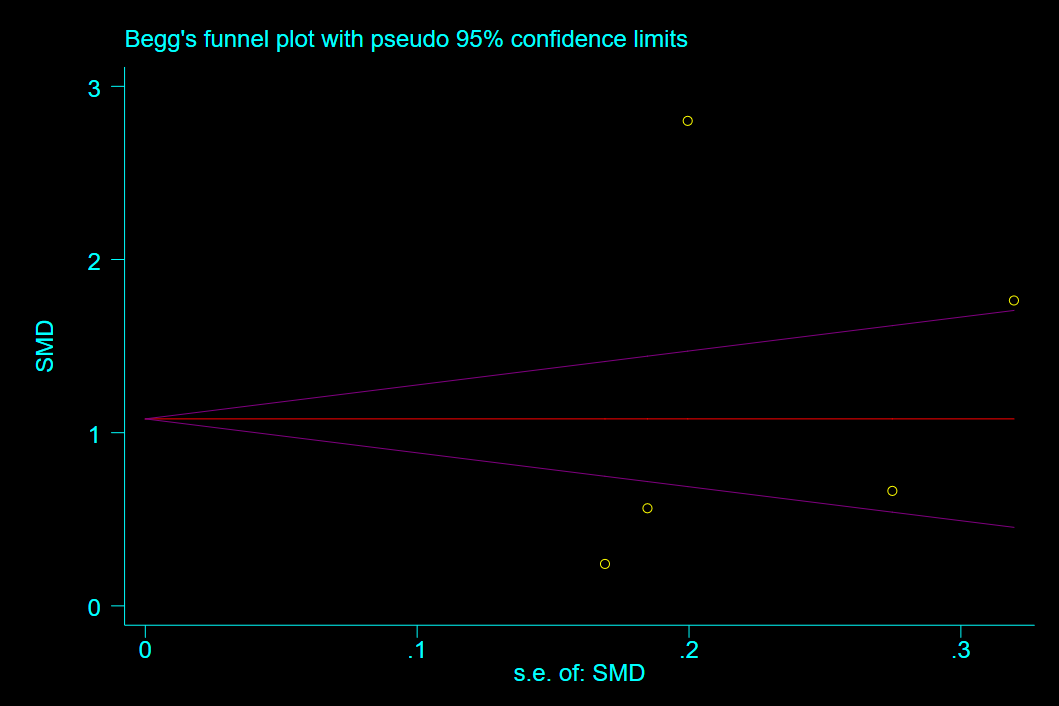


H


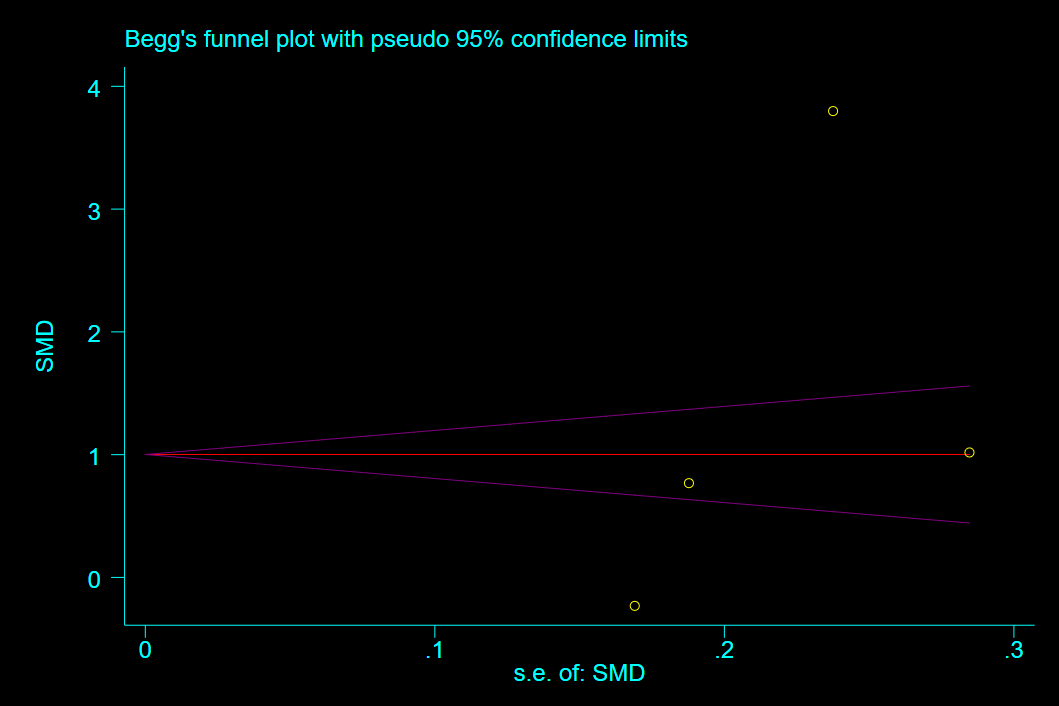


I


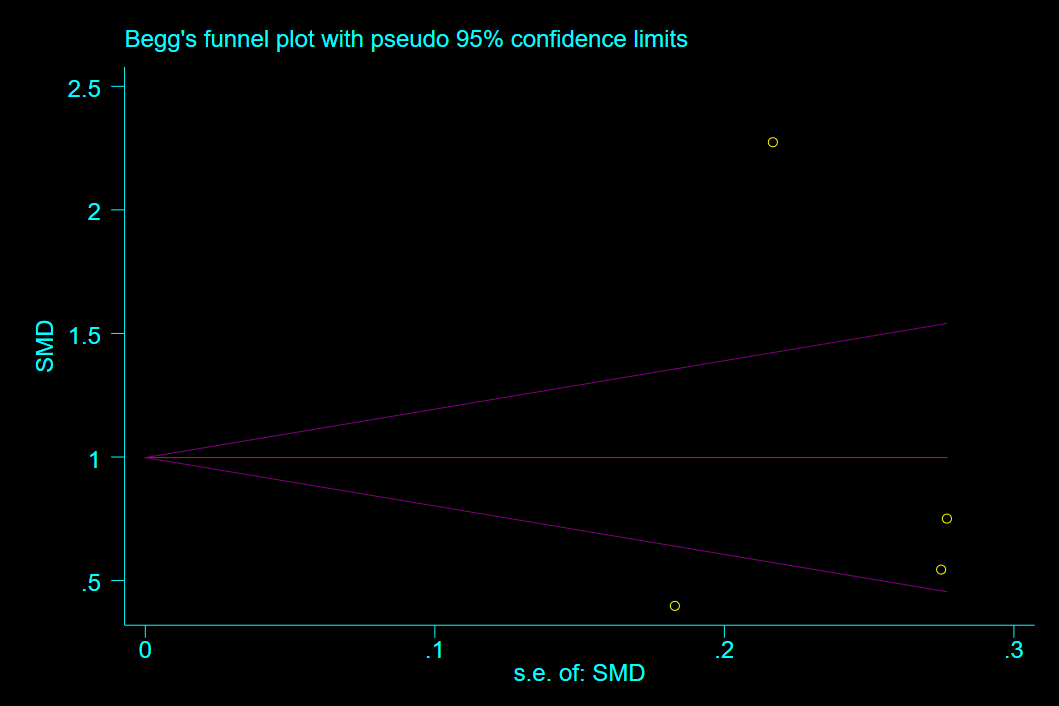


J


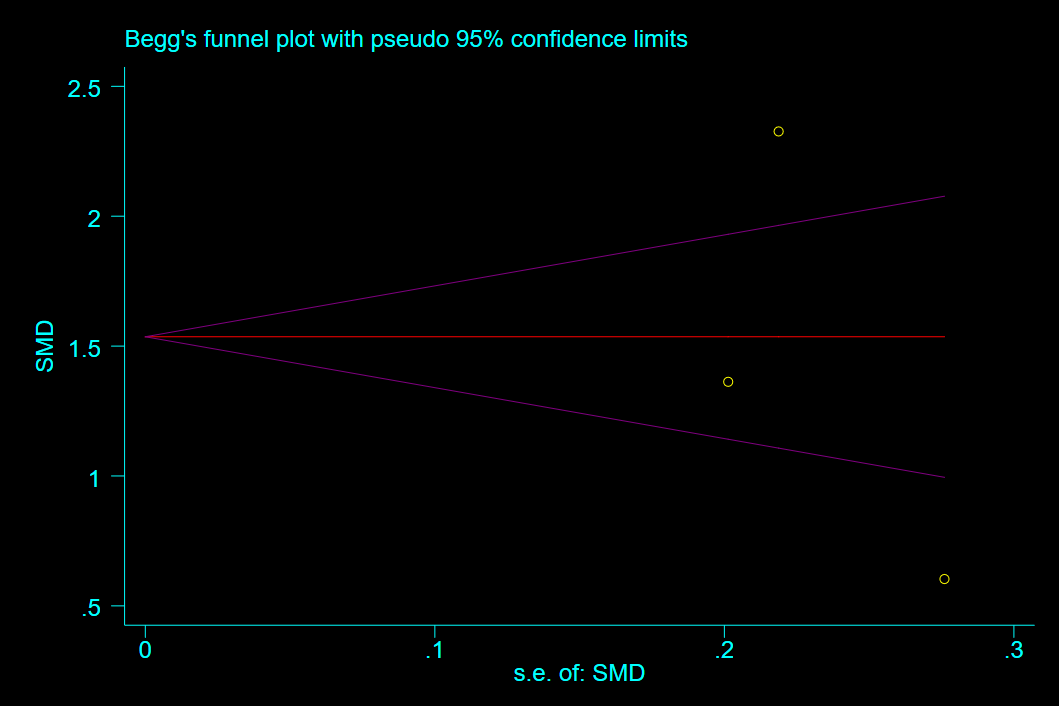

Supplement: Supplementary file 6 [file Table_6.docx]
